# Placental Protein Citrullination Signatures Are Modified in Early- and Late-Onset Fetal Growth Restriction

**DOI:** 10.3390/ijms26094247

**Published:** 2025-04-29

**Authors:** Owen R. Vaughan, Kasia Maksym, Sara Hillman, Rebecca N. Spencer, Mariya Hristova, Anna L. David, Sigrun Lange

**Affiliations:** 1 Department of Maternal and Fetal Medicine, EGA Institute for Women’s Health, University College London, London WC1E 6HX, UK; o.vaughan@ucl.ac.uk (O.R.V.); sara.hillman@ucl.ac.uk (S.H.); r.n.spencer@leeds.ac.uk (R.N.S.); a.david@ucl.ac.uk (A.L.D.); 2 Women’s Health Division, University College London Hospitals NHS Foundation Trust, London NW1 2PG, UK; katarzyna.maksym@nhs.net; 3 Department of Obstetrics and Gynaecology, University of Leeds, Leeds LS2 9JT, UK; 4 Department of Neonatology, EGA Institute for Women’s Health, University College London, London WC1E 6BT, UK; m.hristova@ucl.ac.uk; 5 Pathobiology and Extracellular Vesicles Research Group, School of Life Sciences, University of Westminster, London W1W 6UW, UK

**Keywords:** fetal growth restriction (FGR), placenta, peptidylarginine deiminases (PADs), citrullination/deimination, Histone H3, gene ontology, extracellular vesicle, maternal–fetal, neurodevelopment, inflammation

## Abstract

Fetal growth restriction (FGR) is an obstetric condition most frequently caused by placental dysfunction. It is a major cause of perinatal morbidity with limited treatment options, so identifying the underpinning mechanisms is important. Peptidylarginine deiminases (PADs) are calcium-activated enzymes that mediate post-translational citrullination (deimination) of proteins, through conversion of arginine to citrulline. Protein citrullination leads to irreversible changes in protein structure and function and is implicated in many pathobiological processes. Whether placental protein citrullination occurs in FGR is poorly understood. We assessed protein citrullination and PAD isozyme abundance (PAD1, 2, 3, 4 and 6) in human placental samples from pregnancies complicated by early- and late-onset FGR, compared to appropriate-for-gestational-age (AGA) controls. Proteomic mass spectrometry demonstrated that the placental citrullinome profile changed in both early- and late-onset FGR, with 112 and 345 uniquely citrullinated proteins identified in early- and late-onset samples, respectively. Forty-four proteins were citrullinated only in control AGA placentas. The proteins that were uniquely citrullinated in FGR placentas were enriched for gene ontology (GO) terms related to neurological, developmental, immune and metabolic pathways. A greater number of GO and human phenotype pathways were functionally enriched for citrullinated proteins in late- compared with early-onset FGR. Correspondingly, late-onset but not early-onset FGR was associated with significantly increased placental abundance of PAD2 and citrullinated histone H3, determined by Western blotting. PAD3 was downregulated in early-onset FGR while abundance of PAD 1, 4 and 6 was less altered in FGR. Our findings show that placental protein citrullination is altered in FGR placentas, potentially contributing to the pathobiology of placental dysfunction.

## 1. Introduction

Fetal growth restriction (FGR) is an obstetric condition affecting 3–7% of pregnancies, with higher frequency in low-to-middle-income countries [1,2,3]. To date, clinical management is limited to planning optimal timing of birth and there are no treatments that can improve fetal growth before birth [4]. The aetiology of FGR is often attributed to placental insufficiency [3].

Early-onset FGR (<32 weeks) is more often associated with pre-eclampsia and serious maternal and perinatal adverse outcomes than late-onset FGR (>32 weeks). However, late-onset FGR affects more pregnancies and can be challenging to detect [5]. Both forms of FGR are associated with an increased risk of stillbirth and can lead to poor neonatal and longer-term cardiometabolic and neurodevelopmental outcomes [6,7,8,9]. The underlying mechanisms remain poorly understood. Identifying the molecular pathways underpinning placental alterations is of considerable interest as it could lead to new therapeutic targets.

Peptidylarginine deiminases (PADs) are phylogenetically conserved calcium-activated enzymes, with five human isozymes (PAD1, 2, 3, 4 and 6). PAD isozymes show some differences in tissue-specific expression patterns and in target protein preferences, with some overlap. PAD1 generally associates with epidermis and uterus but also nervous tissue; PAD2 is the most ubiquitously expressed isozyme and detected in most tissues; PAD3 relates to skin, hair follicles and the nervous system; PAD4 mainly to white blood cells and cancers and PAD6 mainly associates with embryonic tissues [10,11,12]. PADs cause post-translational citrullination/deimination in proteins by irreversibly converting arginine into citrulline. This can lead to changes in protein structure and function, including modified protein interactions, loss of function, protein degeneration and neo-epitope formation, which is in turn associated with inflammation [10,11,12,13]. Citrullination can also contribute to protein moonlighting, by allowing different functions of the same protein in physiological and pathobiological processes [14]. PADs are implicated in inflammatory and degenerative diseases including neurodegeneration, autoimmunity, infection and cancers [15,16,17,18,19]. PADs also have roles in development, particularly in embryo pre-implantation and in early embryonic arrest, where the focus has been on roles for PAD6 [20,21,22].

To date, studies on PADs and citrullination in the placenta are limited. PAD1 was identified at the mRNA level in human placentas [23] and PAD1 and PAD2 expressions were quantified in placental cytotrophoblast cells [24,25]. PAD2 was localised to trophoblast cells in placental tissue samples from sheep, horses, dogs and guinea pigs [26], but assessment of all five PAD isozymes in the placenta is lacking to date. Studies are scarce on possible roles for PADs and citrullination in FGR, but there is some evidence that gestational diabetes and pregnancy loss are associated with NETosis (neutrophil extracellular trap formation), which is characterised by histone H3 citrullination and can be partly PAD2 or PAD4-driven, with most studies to date focusing on PAD4 [27,28,29].

The current study aimed to identify citrullinated proteins in human placental samples from both early- and late-onset FGR, in comparison with placentas from appropriate-for-gestational-age (AGA)-term control pregnancies. The five PAD isozymes and histone H3 citrullination were also assessed in the same placental samples by Western blotting. We hypothesised that protein citrullination is altered in the placenta of pregnancies affected by FGR according to the severity and gestational age at onset.

## 2. Results

### 2.1. The Placental Protein Citrullinome Is Modified in FGR Compared with Control AGA Placentas

**Figure 1:**
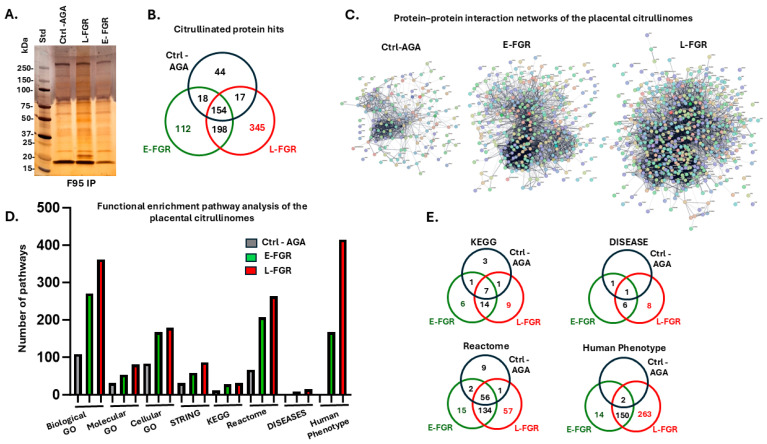
Citrullinated proteins isolated from the control and FGR placental samples. (**A**) A silver-stained protein gel (4–20% TGX gel) showing the pan-citrulline (F95) enriched protein fractions isolated from the three placental groups: control (Ctrl-AGA), late-onset FGR (L-FGR) and early-onset FGR (E-FGR), respectively. (**B**) A Venn diagram summarising the citrullinated protein hits which were identified by LC-MS/MS; shared and unique protein hits are shown for the control and FGR groups (for a full list of protein hits, see Supplementary Table S1). (**C**) Protein–protein interaction (PPI) networks of the placental citrullinomes with individual protein hits represented by the coloured nodes: Ctrl-AGA group (160 nodes, 947 edges); E-FGR group (335 nodes, 3834 edges); L-FGR group (545 nodes, 4771 edges). PPI enrichment *p*-value < 1.0 × 10^−16^ for all. (**D**) Functional enrichment pathway analysis for the placental citrullinomes, comparing control (AGA) and FGR placental samples. The graph compares total numbers of citrullinome-associated pathways identified per group (control, E-FGR, L-FGR), with respect to Biological GO, Molecular GO, Cellular GO, STRING cluster, KEGG, Reactome, DISEASES and Human Phenotype pathways. (**E**) The Venn diagrams summarise shared and unique KEGG, Reactome, DISEASE and Human Phenotype pathways for the citrullinomes of control, E-FGR and L-FGR placental samples. (For full details on the KEGG and DISEASE pathways, see Supplementary Table S2; for Human Phenotypes, see Supplementary Table S3; for Reactome pathways, see Supplementary Table S4).

Citrullinated proteins isolated from control (AGA), early-onset FGR (E-FGR) and late-onset FGR (L-FGR) placentas were separated by SDS-PAGE and silver stained (Figure 1A). Liquid chromatography–mass spectrometry (LC-MS/MS) identified both a greater total number of citrullinated proteins and a greater number of uniquely citrullinated proteins in late- and early-onset FGR placentas, compared to AGA controls (Figure 1B, *p* < 0.001 chi-squared test). The two FGR groups shared more commonly citrullinated proteins than either shared with the control (AGA) group (Figure 1B). The list of citrullinated proteins in each group is given in Supplementary Table S1. The protein–protein interaction networks generated for the placental citrullinomes of the control AGA, E-FGR and L-FGR groups, respectively (Figure 1C; PPI enrichment *p*-value < 1.0 × 10^−16^ for all), reflected the increase in the number of citrullinated protein hits and interactions (number of nodes and expected number of edges) identified in FGR placentas.

In line with the greater number of citrullinated proteins identified, there were more significantly enriched annotation pathways in the FGR placentas compared to controls (Figure 1D). Furthermore, more annotation pathways were significantly enriched in citrullinated proteins in late-onset than in early-onset FGR (Figure 1D). Numbers of shared and unique KEGG, Reactome, DISEASE and Human Phenotype pathways between the placental citrullinomes of the three groups are summarised in Figure 1E, with full lists provided in Supplementary Tables S2–S4. The full LC-MS/MS analysis of citrullinated proteins in the three sample groups is in addition provided in Supplementary Tables S5–S7.

**Figure 2:**
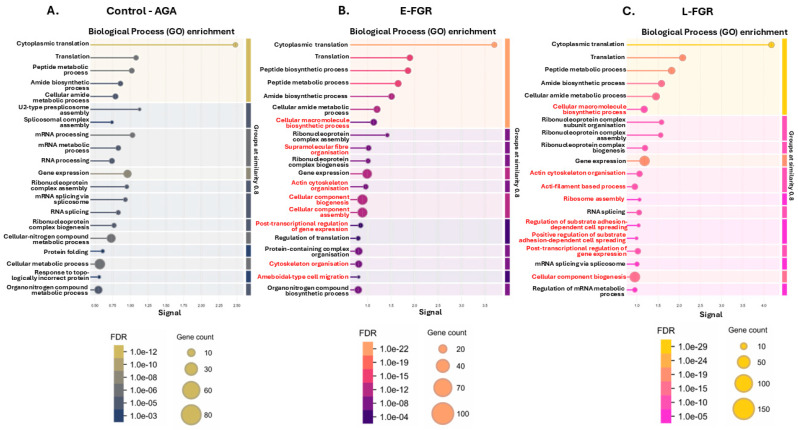
Biological Process (GO) enrichment pathways for the placental citrullinomes. For each group the top 20 pathways are shown: (**A**) control (AGA) placental citrullinome; (**B**) early-onset FGR placental citrullinome; (**C**) late-onset FGR placental citrullinome. The graphs show Signal on the x-axis and false discovery rate (FDR) on the y-axis. Gene count indicates number of proteins associated with the term. Red highlighted terms are unique to the top 20 terms identified for the FGR groups. The red highlighted terms are unique to the respective group.

**Figure 3:**
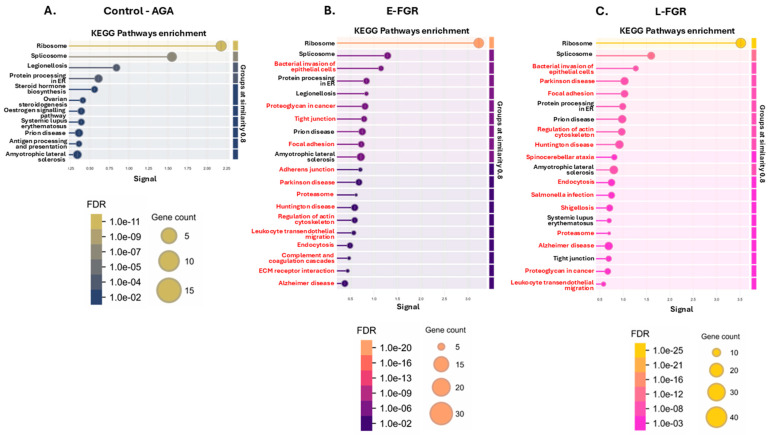
KEGG enrichment pathways. For each group, the top pathways are shown. (**A**) Control (AGA) placental citrullinome had 11 associated KEGG pathways. (**B**) The 20 top KEGG pathways for the early-onset FGR placental citrullinome. (**C**) The 20 top KEGG pathways for the late-onset FGR placental citrullinome. The graphs show Signal on the x-axis and false discovery rate (FDR) on the y-axis. Gene count indicates number of proteins associated with the term. The red highlighted terms are unique to the respective group.

**Figure 4:**
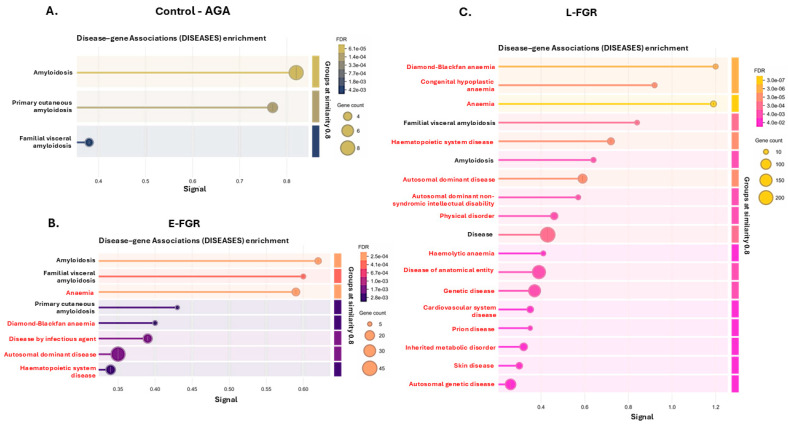
Disease–gene associations (DISEASES) enrichment. In the control group (**A**), three DISEASES pathways were associated with the placental citrullinome, eight pathways were identified for the E-FGR (**B**) and 18 pathways for the L-FGR (**C**) placental citrullinomes. The graphs show Signal on the x-axis and false discovery rate (FDR) on the y-axis. Gene count indicates number of proteins associated with the term. The red highlighted terms are unique to the respective group.

**Figure 5:**
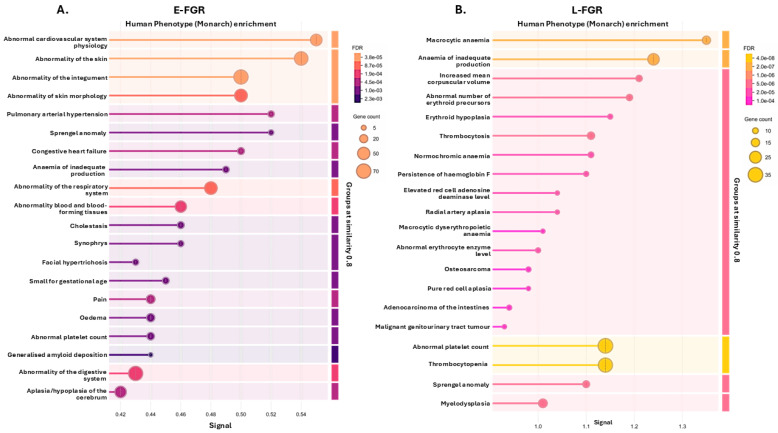
Top 20 Human Phenotype pathways associated with the placental citrullinome of the two FGR groups (**A**) E-FGR and (**B**) L-FGR; none were associated with the control group. The graphs show Signal on the x-axis and false discovery rate (FDR) on the y-axis. Gene count indicates number of proteins associated with the term.

The networks were furthermore compared for the top 20 Biological Process GO pathways, KEGG Pathways, Disease Gene Associations (DISEASES) and Human Phenotype Associations (Monarch) as presented in Figure 2, Figure 3, Figure 4 and Figure 5. Citrullinated proteins were significantly enriched in Biological Process GO terms related to translation and peptide metabolism both in FGR and AGA placentas (Figure 2A–C). By contrast, proteins related to actin cytoskeleton organisation and post-transcriptional regulation of gene expression were citrullinated in FGR but not AGA placentas. Proteins related to cell substrate adhesion were citrullinated only in late-onset FGR.

Similarly, KEGG pathways related to the ribosome and spliceosome were enriched with citrullinated proteins in all three study groups (Figure 3A–C). Actin cytoskeleton-regulating pathways were commonly enriched in both FGR groups but not in the AGA group. Proteins involved in endocytosis were significantly citrullinated only in late-onset FGR placentas. Further pathways specific to the FGR groups related to bacterial invasion, infection and inflammation, cancer, neurodegenerative and neurological diseases (Figure 3B,C; for full details on the KEGG pathways, see Supplementary Table S2).

When comparing enrichment for disease–gene associations (DISEASES), a greater number of terms were enriched with citrullinated proteins in FGR than AGA placentas (Figure 4A–C). Terms enriched in early- and late-onset FGR included haematopoietic system pathology (including Diamond–Blackfan anaemia) and infectious disease. Pathways specific to the L-FGR group included also intellectual disability, cardiovascular system disease, prion disease and inherited metabolic disorder (Figure 5; for full details on DISEASE pathways, see Supplementary Table S2).

Proteins associated with human phenotypic abnormalities of the cardiovascular system, skin, respiratory system, blood forming tissues, digestive system and cerebrum were all citrullinated in FGR placentas (Figure 5A,B). Conversely, citrullinated proteins in the AGA placentas were not enriched in any human phenotypic abnormality terms. A full list of Human Phenotypes identified for the FGR groups is provided in Supplementary Table S3.

### 2.2. Citrullinated Proteins and Functional Enrichment Associations Identified in FGR Placentas Only

**Figure 6:**
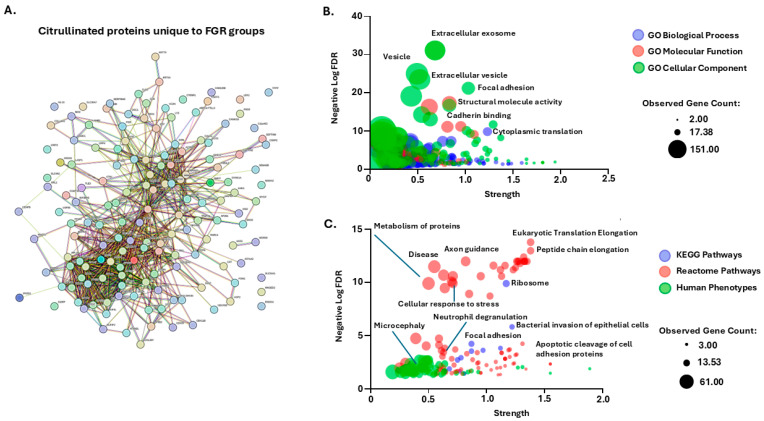
Citrullinated proteins associated with the FGR groups only but not identified in the control (AGA) placentas. (**A**) Protein–protein interaction network (151 nodes, 470 edges, PPI enrichment *p*-value < 1.0 × 10^−16^). (**B**) Enriched GO pathways showing Biological Process, Molecular Function and Cellular Component, with some of the highest enriched terms annotated. (**C**) KEGG and Reactome pathways, alongside Human Phenotype (Monarch) pathways, with selected annotations highlighting some of the top terms identified, based on FDR and strength.

In addition, we assessed protein–protein interaction networks for the citrullinated protein hits which were specific for both FGR groups combined, but were not identified in the control (AGA) group (Figure 6A). Based on FDR and strength, highly enriched GO terms were associated with extracellular vesicles, focal adhesion, cadherin binding, cytoplasmic translation and structural molecule activity (Figure 6B). Top KEGG pathways related to ribosome, bacterial invasion of epithelial cells and focal adhesion (Figure 6C). Top Reactome pathways related to axon guidance, eukaryotic translation elongation, peptide chain elongation, neutrophil degranulation, apoptosis and cellular response to stress (Figure 6C). Top citrullinome-associated Human Phenotypes included microcephaly, immune, cardiovascular, metabolic and digestive abnormalities (Figure 6C).

### 2.3. Placental PAD Isozyme Abundance and Histone H3 Citrullination Are Modified in FGR

**Figure 7:**
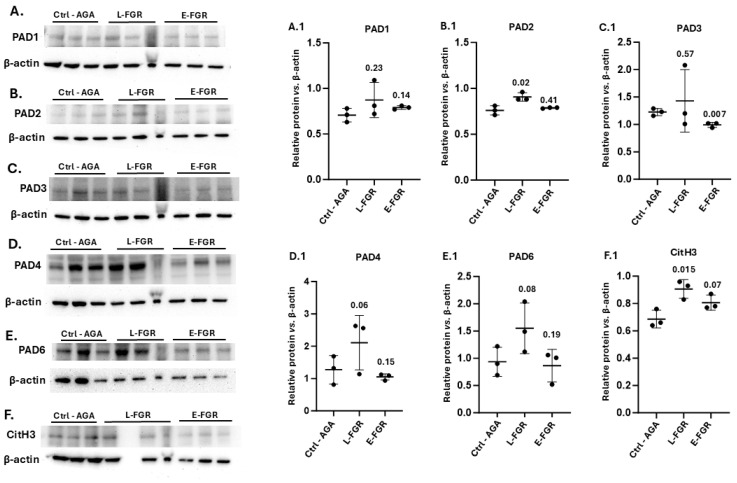
PAD isozyme protein levels and citrullinated histone H3 (CitH3) were assessed by Western blotting comparing appropriate-to-gestational-age (AGA) control and FGR placental samples. (**A**–**E**) Protein detection of PAD1,2,3,4 and 6 is presented, compared with β-actin loading control. (**F**) Histone H3 citrullination (CitH3) is shown in the same samples, compared with β-actin loading control. For each group, 3 × 3 samples were pooled per lane. (**A.1**–**F.1**) Protein densitometry was performed relative to β-actin and is shown in the graphs. Significance was considered at *p* ≤ 0.05; exact *p*-values are shown. Control (Ctrl-AGA), late-onset FGR (L-FGR), early-onset FGR (E-FGR).

When assessing the five human PAD isozymes (PAD1,2,3,4 and PAD6) by Western blotting in the three sample groups, PAD3, 4 and 6 were detected at higher levels, while PAD1 and PAD2 were detected at lower levels, based on the densitometry analysis with beta-actin (Figure 7A–E). Late-onset FGR was associated with significantly increased placental PAD2 abundance, compared to AGA controls (Figure 7B,B.1; *p* = 0.02). Late-onset FGR was also associated with a trend in increased placental abundance of PAD1, PAD3, PAD4 and PAD6 compared to the AGA control group, although this effect was not statistically significant (Figure 7). By contrast, early-onset FGR was associated with a reduced placental PAD3 abundance compared to the AGA control group (Figure 7C,C.1; *p* = 0.007) but showed no significant differences for the other PADs (Figure 7). Since LC-MS/MS identified histone H3 amongst the citrullinated proteins in late-onset FGR placentas, we further assessed changes in citrullinated histone H3 (CitH3) abundance by Western blotting. CitH3 levels were significantly elevated in the late-onset FGR group (*p* = 0.015) but not in the early-onset FGR group (*p* = 0.07), compared with AGA controls (Figure 7F,F.1).

## 3. Discussion

### 3.1. Possible Roles for Citrullination in Placental Function and Implications in FGR

Proteins involved in translation, peptide metabolism and ribosomal function were highly citrullinated in all placentas, consistent with the observation that citrulline bioavailability regulates protein synthesis in skeletal muscle [30]. The specific pathways enriched depended on the gestational age at FGR diagnosis. In the placenta, a high rate of protein synthesis is required for expansion of the syncytiotrophoblast epithelium, maintenance of the nutrient transport apparatus and hormone production [31]. Placental protein synthesis is regulated by environmental signals like oxygen tension and impaired in pregnancies complicated by FGR [32,33,34]. Citrullination may therefore contribute to regulating protein synthesis in the placenta, via effects on ribosomal translation.

In line with the association between FGR and citrullination of proteins related to the actin cytoskeleton, actin and tubulin citrullination is implicated in modulating microtubule dynamics in hormone-secreting cells [35]. In the placenta, cytoskeletal proteins regulate syncytiotrophoblast nutrient transport by mediating transporter trafficking to the microvillous plasma membrane [36] and maintain the architecture and polarity of the epithelium itself [37]. They are known to be altered in function in placentas from pregnancies complicated by FGR [37]. Cytoskeletal protein citrullination could therefore underpin impaired nutrient transport and altered microvillous ultrastructure in FGR.

As extracellular vesicles (EVs) play crucial roles in cellular communication in health and disease, the identification of EV pathways for the FGR citrullinome is furthermore of interest for placental function and maternal–fetal crosstalk [38,39,40,41,42,43,44,45,46]. EV release profiles and EV cargoes are modified by gestational diseases [39,40,41,42]. In FGR, changes in placental-derived EV ratios have been reported [43] and, in maternal plasma from FGR pregnancies, reduced EV concentrations with modified inflammatory protein cargo profiles have been observed [44]. Placenta-derived EVs from pre-eclamptic pregnancies have been shown to negatively affect vascular endothelial function [45,46]. Trophoblast-derived EVs can promote pre-eclampsia by regulating macrophage polarization [41], while macrophage-derived EVs have been shown to have roles in spontaneous abortion by affecting trophoblast migration and invasion [47]. Placental-derived EVs are therefore receiving increasing attention as biomarkers for monitoring pregnancy and pregnancy complications [42,48,49,50]. As modulating roles for PADs in EV biogenesis via actin and PAD-mediated effects on cargo signatures have been reported in various disease models [51,52,53,54,55,56,57], the citrullinome changes identified here in FGR may affect EV-mediated maternal–fetal communication, with possible consequences for adverse outcomes.

Developmental pathways identified for both FGR placental citrullinomes included WNT signalling and Hedgehog Reactome pathways as well as NOTCH signalling. WNT and NOTCH are critical pathways in human placenta and trophoblast development [58] and in placental angiogenesis, where Hedgehog also plays important roles. Citrullination in these pathways is therefore of considerable interest, as they are important for placental function and have been identified to be relevant to FGR scenarios [59].

The specific association between late-onset FGR and citrullination of proteins related to cell adhesion may suggest that the invasive and migratory properties of trophoblast cells are affected by this post-translational modification. Citrullination of extracellular matrix proteins like collagen and fibronectin generally increases invasive behaviour of fibroblasts in vitro [60,61] and cell adhesion influences trophoblast invasion, determining placental function [62]. Late-onset FGR has also been associated with citrullination of proteins involved in endocytosis, which is a key determinant of placental transfer, particularly of antibodies [63], although there is limited information on its regulation by citrullination. Increased PAD2 abundance in late-onset FGR placentas is consistent with the broad tissue distribution of this isozyme and its reported upregulation in other diseases, including several neurodegenerative conditions and cancers [64,65,66]. Our data may therefore indicate a mechanism whereby PAD2 upregulation contributes to placental insufficiency in late-onset FGR by altering trophoblast function. Previously, PAD2 and PAD3 have been linked to regulation of trophoblast stem cell differentiation, via DNA methylation [67].

### 3.2. Placental Citrullinome Associations to Disease and Phenotype Pathways in FGR

By comparing our results from the functional pathway enrichment analysis for the placental citrullinomes to FGR-associated abnormalities and pathologies, we may gain some insights into putative roles for citrullination in adverse outcomes.

Early-onset FGR associates with fetal hypoxia, fetal demise and preterm delivery. Morbidities related to prematurity can also occur, including necrotising enterocolitis, intracranial haemorrhage and retinopathy [68]. This relates to some of the unique E-FGR citrullinome-associated Human Phenotypes identified here, which included premature birth, dysplastic corpus callosum, abnormal adipose tissue morphology, muscle fibre atrophy, hypotension and abnormality of cranial sutures (Supplementary Table S3). PADs and citrullination have previously been reported to be important players in hypoxic ischaemic brain insult and neonatal seizures [69,70,71]. Furthermore, PAD6 is associated with pre-implantation, roles in early development, early embryonic arrest and miscarriage [20,21,22].

Late-onset FGR is a common cause of stillbirth and furthermore leads also to poor long-term cardiometabolic and neurodevelopmental neonatal outcomes [6,7,9,72]. This correlates with some of the unique citrullinome DISEASES pathways identified for the L-FGR group, including cardiovascular system disease, metabolic disorder and intellectual disability. Terms related to reported physical signs in growth-restricted infants were also identified, including low-set ears, hand abnormalities, cleft palate, scalp defect, abnormal eye and micrognathia [73]. The 263 Human Phenotype terms identified as unique to the L-FGR citrullinomes included glaucoma, musculoskeletal abnormalities, neoplasms, leukaemia, gastrointestinal problems, chronic infection, kidney and renal disease. These findings show some differences in citrullinome profiles based on the severity of FGR, which is directly related to perinatal mortality and risk of longer-term adverse outcomes. It is known that FGR can lead to cognitive and neurodevelopmental abnormalities [2] and an increased risk of metabolic, cardiovascular and renal diseases later in life [74,75,76].

Additional shared pathways enriched for both the E-FGR and the L-FGR placental citrullinomes (but not detected in the control placental citrullinome) consolidated associations with neurodevelopmental and metabolic abnormalities, and muscle-associated, cardiac, digestive and inflammatory pathways. Citrullinome-enriched pathways also related to size for gestational age, growth delay, microcephaly, eye, kidney and renal abnormalities. Protein misfolding and defective amyloid processing, which is associated with pre-eclampsia [77], was linked to Reactome and Disease pathways both in E-FGR and L-FGR placentas. Alterations in ribosome pathways were identified for both FGR citrullinomes and are linked to ribosomepathies, including rare diseases and Diamond–Blackfan anaemia [78]. Interestingly, Diamond–Blackfan anaemia was diagnosed in one of the E-FGR affected babies of the EVEREST study cohort [8].

The citrullinome-associated link to several infection pathways in the placental citrullinomes of the FGR groups may be of interest, as fetal infection is responsible for 5–10% of FGR cases [3]. Furthermore, sepsis and immunological incompetence are reported [74,79,80]. *S. aureus* infection has been shown to pass through the placental barrier and affect fetal growth and development [81], while sepsis is reported in very preterm infants [82,83] and linked to stillbirth due to fetal invasive infections [84]. Salmonella infection can induce altered placental morphometrics and adverse pregnancy outcomes [85,86]. Shigellosis can result in miscarriage or preterm birth [87,88] and Yersenia infection has been linked to fetal death and pregnancy course disorders, with possible implications in FGR [89,90]. PADs are known to have critical roles in infection, with effective PAD inhibitor treatment in sepsis and endotoxemia including by inhibition of NETosis [91,92,93] and effects on bacterial EV release and antibiotic treatment sensitisation [94].

It has also been suggested that early life events such as intrauterine growth retardation may affect immune function in the longer term, including autoimmune diseases [95,96]. Some associations to chronic and autoimmune disease were identified here for the placental citrullinomes, including cytokine signalling for the Reactome of both FGR groups and the lupus KEGG pathway for the L-FGR group. Furthermore, recent studies have highlighted long-term neurological effects due to FGR, including impaired cognitive outcomes and dementia with ageing in affected individuals [97,98]. This links to several neurodegenerative disease associations with the placental citrullinomes of the FGR groups and to reported roles for citrullination in several neurodegenerative diseases [15,66,99]. It can be postulated that placental citrullinome changes in FGR may be an epigenetic contributor to longer-term chronic and degenerative diseases.

In addition to associations of the placental citrullinome to fetal outcomes, the link to maternal effects must also be considered. Adverse effects of maternal autoimmune diseases on pregnancy outcomes have been reported, including systemic lupus erythematosus, Sjögren’s syndrome, rheumatoid arthritis, type 1 diabetes, coeliac disease and thyroid autoimmunity [100], all of which have been associated with changes in citrullination [18,101,102]. To what extent maternal citrullinated auto-antibodies may contribute to adverse outcomes in FGR will need further investigation and validation as this is currently understudied. Maternal hypothyroidism and hypertension are both associated with an increased risk of FGR [103], and interestingly thyroid hormone synthesis was a KEGG pathway identified in the E-FGR placental citrullinome while hypertension was associated with the E-FGR and L-FGR placental citrullinomes. Abnormal maternal liver function is also reported in FGR [104]. Abnormality of the liver was here identified as a Human Phenotype pathway for the L-FGR placental citrullinome, but abnormal liver function usually arises from pre-eclampsia which is more common in E-FGR. Our findings indicate that changes in placental citrullination in FGR may be associated with both fetal and maternal effects, and lays foundations for further in-depth investigations for PADs in FGR.

### 3.3. Histone H3 Citrullination in FGR

Citrullinated histone H3 levels were assessed in the placental samples by Western blotting, using the anti-histone H3 (citrulline R2 + R8 + R17) antibody commonly used for assessment of NETosis. CitH3 levels were significantly elevated in the L-FGR group. This did link to the significant increase in PAD2, and there was also a trend (albeit non-significant) for increased PAD4 in the L-FGR placentas. Both PAD2 and PAD4 have been linked to PAD-driven NETosis in experimental and clinical studies [105,106]. The change in CitH3 abundance observed by Western blotting correlated with the LC-MS/MS analysis, where CitH3 was amongst the citrullinated protein hits in the L-FGR group. There is an increased interest in epigenetic effects in FGR, including DNA methylation status and post-translational modifications of histones [107,108]. Epigenetic DNA modifications have been associated, for example, with metabolic syndrome [109]. Roles for post-translational modifications of histones have received interest in placenta relating to pre-eclampsia [110] and in transgenerational impacts and fetal programming [111,112]. Histone H3 modifications have been reported in human placentas affected by FGR, with possible sex-specific differences [113], but current understanding of histone H3 citrullination in FGR is still limited. NETosis can be identified by CitH3 labelling and has been reported to be increased in neutrophils from gestational diabetes mellitus [114] and suggested to have roles in pre-eclampsia and fetal loss [115]. Furthermore, a recent study quantified CitH3 by ELISA as a marker of NETs in placental neutrophils in HIV infection and pre-eclampsia [116]. This correlates with our finding that the E-FGR and L-FGR placental citrullinomes related to Reactome pathways for Host Interactions of HIV factors and HIV infection, respectively. The Reactome analysis also identified SARS-CoV infection pathways for the placental citrullinomes, and these have been associated to PADs and NETosis [117,118], also with possible implications in FGR, including placental abnormalities [119,120,121,122]. A mouse model of antiangiogenic factor-mediated pregnancy loss showed a relationship between PAD4 knockout and reduced NETs (assessed by CitH3 staining), which resulted in fewer inflammatory responses and reduced pregnancy loss [27]. Placentas from pre-eclamptic compared to non-hypertensive pregnant women showed increased NETs and CitH3 staining, supporting roles for proinflammatory and procoagulant effects of NETs [27]. In a study inducing gestational diabetes mellitus in PAD4-deficient mice, the placental weight of the PAD4 KO mice increased significantly, indicating that the inhibition of NET formation increased placental and fetal weight, without affecting placental structure [28]. At this stage, we can postulate that both PAD2 and PAD4 cause histone H3 citrullination in the placental samples, although it will need further validation, including using in vitro trophoblast PAD isozyme specific knockout models. The epigenetic effects of histone citrullination and effects of specific PAD isozymes in epigenetic regulation remain to be further studied in FGR. Histone tails are inherently disordered structures, which makes them highly susceptible to post-translational modifications, including citrullination, playing important roles in transcriptional regulation [123]. Accumulating research, including our findings here, will increase current understanding of the effects of histone citrullination in FGR, both with respect to epigenetic modulation as well as inflammatory responses via NETosis.

This pilot study is based on a small sample number and is vulnerable to the usual limitation of placental research that “healthy” control samples are only available at term, resulting in a significant difference in gestational age from E-FGR placentas. However, this is balanced by the detailed phenotyping of the pregnancies, including complete follow-up, prospective sample collection according to a defined protocol and incorporating a balance of placentas from male and female fetuses. While outside of the scope of our current study, the assessment of sex-specific differences, including placentas from larger sample groups of male and female fetuses, respectively, per group, may be of considerable interest for the identification of putative sex-specific differences for future studies. The citrullinome protein–protein interaction networks are based on known and predicted interactions which are experimentally determined, from curated databases, or based on gene neighbourhood, gene fusions and gene co-occurrence, in addition to text mining, co-expression and protein homology. Our results provide the first representative placental citrullinome for early- and late-onset FGR with insights into possible roles for citrullination in placental function and pathogenesis of FGR. This will aid future in-depth studies on roles for this post-translational modification and possible therapeutic avenues in FGR.

## 4. Materials and Methods

### 4.1. Samples and Sample Preparation

Placental villous tissue was obtained with written, informed consent under studies ethically approved by a UK National Health Service Research Ethics Committee (Stanmore, 13/LO/1254 or Hampstead, 15/LO/1488). Pregnant women with early-onset FGR were recruited to the study between 20 + 0 and 26 + 6 weeks’ gestation, as previously described [124]. They were diagnosed based on ultrasound estimated foetal weight below 600 g and <3rd centile for gestational age. Pregnant women with late-onset FGR and with appropriate-for-gestational-age (AGA) control babies were recruited when they delivered at term. Late-onset FGR was diagnosed if the fetus was appropriately grown with EFW > 10th centile for gestational age at the mid-gestation anomaly scan (19–21 weeks’ gestation) but had an EFW < 10th centile after 32 + 0 weeks of gestation. Exclusion criteria for all groups included multiple pregnancy, maternal age < 18 years, fetal structural or karyotypic abnormalities or maternal virus infection. Placental villous tissue was systematically sampled from the placental parenchyma, midway between the umbilical cord insertion and margin within 30 min of birth. Samples were dissected free from the decidua and chorionic plate, frozen and stored at −80 °C. For the three groups assessed, both male and female placentas were included (AGA control 5 male, 4 female; early-onset FGR 4 male, 5 female; late-onset FGR 5 male, 3 female); see Table 1.

**Figure 8:**
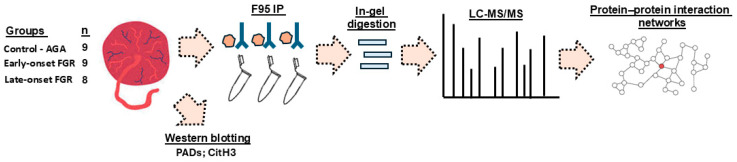
Overview of the experimental setup. Placental samples from control (AGA), early-onset and late-onset FGR groups were used for protein isolation. Proteins were assessed by Western blotting, and immunoprecipitation of citrullinated proteins was carried out with the pan-citrulline F95 antibody. Citrullinated protein fractions were subjected to in-gel digestion and protein hits were identified by LC-MS/MS analysis. The placental citrullinomes were then assessed for protein–protein interaction networks and associated functional enrichment pathway analysis.

Proteins were extracted from individual placental specimens by homogenising them in RIPA+ buffer (Sigma-Aldrich, Gillingham, UK, containing 10% protease inhibitor cocktail, Sigma-Aldrich) in 2 mL Eppendorf tubes on ice, using a Mini Handheld Homogeniser (Kimble, DWK Life Sciences, VWR International, Stoke-on-Trent, UK). For each 100 mg of tissue, 500 µL of RIPA+ buffer was used. The tissue homogenates were incubated for 2.5 h on a continuously rotating platform at 4 °C. Tissue lysates were gently pipetted up and down at regular intervals, using 200 µL pipette tips and a Gilson pipette. Tissue homogenates were then centrifuged at 16,000× *g* for 30 min at 4 °C and the protein-containing supernatant was collected, aliquoted and immediately frozen at −80 °C until further use for Western blotting, immunoprecipitation and LC-MS/MS. The experimental setup is shown in Figure 8.

### 4.2. Isolation of Citrullinated Proteins from Placental Samples

Citrullinated proteins were isolated from the extracted protein lysates by immunoprecipitation using the F95 pan-citrulline antibody (MABN328, Merck, Watford, UK). Protein extracts were pooled from all specimens in each study group (10 µL from each individual sample, a pool of 9 × 10 µL in control, 9 × 10 µL in early-onset FGR and 8 × 10 µL in late-onset FGR groups). Immunoprecipitation was carried out using the Catch and Release^®^ v2.0 Immunoprecipitation Kit (17-500M, Merck) together with the F95 pan-citrulline antibody and the affinity ligand provided with the kit, according to the manufacturer’s instructions (Merck). Immunoprecipitation was performed overnight, incubating the mini-IP columns at 4 °C on a rotating platform. Thereafter the citrullinated (F95 bound) proteins were eluted according to the manufacturer’s instructions (Merck) and assessed by SDS-PAGE and silver staining (BioRad Silver Stain Plus Kit, BioRad, Watford, Hertfordshire, UK) for protein yield and by LC-MS/MS analysis for identification of citrullinated protein hits, per group.

### 4.3. Liquid Chromatography with Tandem Mass Spectrometry (LC-MS/MS)

For LC-MS/MS, the total F95-enriched eluates from each placental sample group were run 0.5 cm into a 12% TGX gel (BioRad) and cut out as one gel band per group. Following in-gel digestion, LC-MS/MS was performed (Cambridge Proteomics, Cambridge, UK), according to previously described methods [55,99,125]. In brief, automated LC-MS/MS analysis was carried out using a Dionex Ultimate 3000 RSLC nanoUPLC system (Thermo Fisher Scientific Inc., Waltham, MA, USA) in conjunction with a QExactive Orbitrap mass spectrometer (Thermo Fisher Scientific Inc., Waltham, MA, USA). Separation of peptides was performed by reverse-phase chromatography at a flow rate of 300 nL/min and a Thermo Scientific reverse-phase nano Easy-spray column (Thermo Scientific PepMap C18, 2 mm particle size, 100A pore size, 75 mm i.d. × 50 cm length). Peptides were loaded onto a pre-column (Thermo Scientific PepMap 100 C18, 5 mm particle size, 100A pore size, 300 mm i.d. × 5 mm length) from the Ultimate 3000 autosampler with 0.1% formic acid for 3 min at a flow rate of 15 mL/min. After this period, the column valve was switched to allow elution of peptides from the pre-column onto the analytical column. Solvent A was water + 0.1% formic acid and solvent B was 80% acetonitrile, 20% water + 0.1% formic acid. The linear gradient employed was 2–40% B in 40 min. Further wash and equilibration steps gave a total run time of 60 min. The LC eluent was sprayed into the mass spectrometer using an Easy-Spray source (Thermo Fisher Scientific Inc.). The *m*/*z* values of all eluting ions were measured in an Orbitrap mass analyser and data-dependent scans (selecting top 20) were employed for automatic isolation and generation of fragment ions using the HCD collision cell, measured using the Orbitrap analyser (ESI-ORBITRAP-HCD). Both singly charged ions as well as ions with unassigned charge states were excluded from selection for MS/MS. A dynamic exclusion window of 20 s was applied. The data were processed post-run using Protein Discoverer (version 2.1., Thermo Scientific), converted to mgf files and submitted to Mascot (Mascot search algorithm; Matrix Science, London, UK). Searching for hits was carried out against the UniProt database Homo_sapiens_20221011 (226,953 sequences; 74,609,178 residues) with peptide and fragment mass tolerances, respectively, set at 20 ppm and 0.1 Da. The threshold value for significance was set at *p* < 0.05, and the peptide cut-off score was set at 38.

### 4.4. Functional Enrichment and Protein–Protein Interaction (PPI) Network Analysis

The list of citrullinated protein hits from the three placental sample groups was functionally annotated and protein–protein interaction networks were constructed using STRING analysis (Search Tool for the Retrieval of Interacting Genes/Proteins; https://string-db.org/, accessed on 20 November 2024). Data for the pathway analysis of the protein networks were exported as STRING network images. Excel files were exported for gene ontology (GO Biological process, Molecular function and Cellular component), Kyoto Encyclopedia of Genes and Genomes (KEGG), Reactome, Disease–Gene associations (DISEASES) and Human Phenotype (Monarch) pathway enrichment analyses.

### 4.5. Western Blotting

For Western blotting, 40 µL aliquots of protein extract per sample were diluted with 40 µL 2× reducing Laemmli sample buffer (BioRad; containing 5% β-mercaptoethanol, Sigma-Aldrich) and boiled for 5 min at 100 °C. Samples were run by SDS-PAGE (4–20% TGX gels, BioRad, Watford, UK) at 165 V for 52 min, using a 5 µL aliquot per sample, per lane. For each group (control AGA, early-onset FGR, late-onset FGR), three individual placenta samples were pooled per lane so that there were three pools representing each group. Proteins were transferred to nitrocellulose membranes using semi-dry transfer (1 h at 15 V), with even protein transfer assessed by PonceauS red stain (Sigma-Aldrich) before blocking in 5% bovine serum albumin (BSA, Sigma-Aldrich) in TBS-T for 1 h at room temperature (RT). For the detection of PAD isozymes, the membranes were incubated in primary antibodies overnight at 4 °C on a shaking platform using the following anti-human PAD isozyme specific antibodies: PAD1 (ab181762, Abcam Cambridge, UK), PAD2 (ab50257), PAD3 (ab50246), PAD4 (ab50247) and PAD6 (PA5–72059, Thermo Fisher Scientific, Hemel Hempstead, UK). Citrullinated histone H3 was detected in the same samples using anti-citH3-r2-r8-r17 (ab5103, Abcam). All primary antibodies were used at 1/1000 dilution in TBS-T as per the manufacturer’s instructions. Following incubation with the primary antibodies, washing was carried out at room temperature (RT) with TBS-T (3 × 10 min). Secondary antibody incubation was completed for 1 h at RT using HRP-labelled anti-rabbit IgG (BioRad, diluted 1/3000 in TBS-T). Following washing (5 × 10 min in TBS-T), visualisation was carried out using enhanced chemiluminescence (ECL, Amersham Biosciences, Buckinghamshire, UK) and the UVP BioDoc-ITTM System (Thermo Fisher Scientific, Dartford, UK). All blots were re-probed with an HRP-conjugated anti-β-actin antibody (ab20272, Abcam, 1/5000 in TBS-T) for quantitative analysis of PAD isozymes and citH3, by protein densitometry analysis, which was carried out using ImageJ (version 1.54h).

### 4.6. Statistical Analysis

For comparison of datasets from the three study groups, GraphPad Prism version 10 was used. One-way ANOVA was used to analyse continuous clinical data and densitometry readings from Western blotting analysis. Categorical clinical data were analysed by Fisher’s exact test. Numbers of citrullinated protein hits in each study group were compared with expected values, assuming equal distribution using the chi-squared test. Statistical significance was regarded as *p* < 0.05. STRING analysis was carried out with medium confidence (https://string-db.org/, accessed on 20 November 2024).

## 5. Conclusions

In summary, our findings show that protein citrullination increases in the placenta in pregnancies complicated by FGR. Protein citrullination may contribute to FGR by altering the function of proteins that regulate placental development, trophoblast invasion or nutrient transport, as well as proteins involved in numerous human disease and phenotype pathways linked to FGR. The results demonstrate that different proteins undergo post-translational citrullination in the placenta in early- and late-onset FGR. The study has major implications for our understanding of the pathogenesis of FGR. Given the potential therapeutic application of PAD inhibitors in other disease areas [10,18,126], the findings may ultimately lead to interventions that mitigate placental insufficiency and benefit pregnant women and babies.

## Figures and Tables

**Table 1:** Clinical characteristics of study participants. Continuous variables are mean ± SD, effect of group determined by one-way ANOVA. Superscripts a,b,c represent significant intergroup differences by Tukey’s post hoc. Categorical variables are number (%); effect of group determined by Fisher’s exact test.

	Control	n	Early-Onset FGR	n	Late-Onset FGR	n	*p* Value
**Ethnicity (white)**	7 (78%)	9	5 (56%)	9	3 (38%)	8	0.291
**Age (year)**	35.8 ± 4.6	9	31.8 ± 4.6	9	31.0 ± 5.7	8	0.119
**Body mass index (kg/m^2^)**	25.4 ± 5.1	9	29.7 ± 12.7	9	27.4 ± 8.2	8	0.627
**Gestation at delivery (wk)**	39.4 ± 0.8 ^a^	9	30.8 ± 5.2 ^b^	9	37.4 ± 1.7 ^a^	8	<0.001
**Delivery mode (c-section)**	8 (89%)	9	9 (100%)	9	7 (88%)	8	0.751
**Infant sex (female)**	4 (44%)	9	5 (56%)	9	3 (38%)	8	0.885
**Birth weight (g)**	3411 ± 386 ^a^	9	1040 ± 679 ^b^	9	2151 ± 467 ^c^	8	<0.001
**Placenta weight (g)**	472 ± 44 ^a^	5	219 ± 108 ^b^	4	330 ± 84 ^b^	6	0.002

^a,b,c^ Superscripts represent significant intergroup differences by Tukey’s post hoc. Continuous variables are mean ± SD; effect of group determined by one-way ANOVA. Categorical variables are number (%); effect of group determined by Fisher’s exact test.

## Data Availability

The data are contained within the article and Supplementary Materials.

## Supplementary Materials

The following supporting information can be downloaded at: https://www.mdpi.com/article/10.3390/ijms26094247/s1.
