# Supplementary material for: Placental Protein Citrullination Signatures Are Modified in Early- and Late-Onset Fetal Growth Restriction
Source: Int J Mol Sci. 2025 Apr 29;26(9):4247. doi: 10.3390/ijms26094247 (PMC12071715; doi:10.3390/ijms26094247)
Supplement: Supplementary file 1 [file ijms-26-04247-s001.zip › Supplementary Table S1.docx]

**Supplementary Table S1.** Citrullinated protein hits identified in control (AGA), early (E-FGR) and late (L-FGR) onset fetal growth restriction (FGR). A tick (V) indicates that the protein was identified in the respective group.

| **Protein ID** | **Protein name** | **Control AGA** | **E-FGR** | **L-FGR** |
| --- | --- | --- | --- | --- |
| A2MG | Alpha-2-macroglobulin | V | V | V |
| A0A384MQX1 | Epididymis secretory sperm binding protein | V | V | V |
| LMNA | Isoform C of Prelamin-A/C | V | V | V |
| A0A024RBH2 | Cytoskeleton-associated protein 4, isoform CRA_c | V | V | V |
| H6VRG0 | Keratin, type II cytoskeletal 1 | V | V | V |
| DHX15 | ATP-dependent RNA helicase DHX15 | V | V | V |
| A0A024R2A7 | Lectin, mannose-binding, 1, isoform CRA_b | V | V | V |
| K2C8 | Isoform 2 of Keratin, type II cytoskeletal 8 | V | V | V |
| A0A384N6C7 | Epididymis secretory sperm binding protein | V | V | V |
| D9YZU5 | Beta-globin | V | V | V |
| A0A3B3ITJ4 | Heterogeneous nuclear ribonucleoprotein L (Fragment) | V | V | V |
| K2C5  P13647 | Keratin, type II cytoskeletal 5 | V | V | V |
| B3VL17 | Beta globin (Fragment) | V |  | V |
| B3VL31 | Beta globin (Fragment) |  | V |  |
| Q4TWB7 | Beta globin |  | V |  |
| A0A384MEE7 | Epididymis secretory sperm binding protein | V | V | V |
| A0A384N5Z8 | Epididymis secretory sperm binding protein | V | V | V |
| TPR | Nucleoprotein TPR | V | V | V |
| D3GKD9 | G-gamma globin Paulinia variant | V | V | V |
| A0A2R8Y7X9 | GLOBIN domain-containing protein | V | V | V |
| A0A384N5Z8 | Epididymis secretory sperm binding protein | V | V | V |
| A0A384N6H1 | Epididymis secretory sperm binding protein |  | V | V |
| A0A384P5K2 | Epididymis secretory sperm binding protein |  | V | V |
| S6B291 | IgG H chain | V | V | V |
| S6BGE0 | IgG H chain |  | V | V |
| Q6J1Z7 | Hemoglobin beta (Fragment) | V | V | V |
| A0N071 | Delta globin | V | V | V |
| FHL1 | Isoform 5 of Four and a half LIM domains protein 1 | V | V | V |
| A0A087WT78  A0A7P0TAE1  HSP90B1 | Endoplasmin | V | V | V |
| SF3B1 | Splicing factor 3B subunit 1 | V | V | V |
| K2C7 | Keratin, type II cytoskeletal 7 | V | V | V |
| K1C19  P08727 | Keratin, type I cytoskeletal 19 | V | V | V |
| A0A0K2BMD8  HBA2 | Mutant hemoglobin alpha 2 globin chain | V | V | V |
| V9HWB4 | 78 kDa glucose-regulated protein | V | V | V |
| V9HW22 | Epididymis luminal protein 33 | V | V | V |
| B4DV94 | cDNA FLJ58285, highly similar to Homo sapiens pre-B-cell leukemia transcription factor interacting protein 1 (PBXIP1), mRNA | V | V | V |
| B4E0K4 | cDNA FLJ58659, highly similar to Homo Sapiens pre-B-cell leukemia transcription Factor interacting protein 1 (PBXIP1), mRNA |  | V |  |
|  |  |  |  |  |
| K1C14 | Keratin, type I cytoskeletal 14 | V | V | V |
| A0A024RAY2 | Keratin 18, isoform CRA_a | V | V | V |
| K1C16 | Keratin, type I cytoskeletal 16 | V | V | V |
| D3DNU8 | Kininogen 1, isoform CRA_a | V | V | V |
| A0A384NYT8 | Tubulin beta chain (Fragment) | V | V | V |
| A0A0K0K1H8 | Serotransferrin | V |  |  |
| B4E1B2 | Serotransferrin |  | V | V |
| A0A024R663 | Kinectin 1 (Kinesin receptor), isoform CRA_a | V |  |  |
| A0A0C4DGN6 | ARF GTPase-activating protein GIT1 | V | V |  |
| Q9Y2X7-3  GIT1 | Isoform 3 of ARF  GTPase-activating protein GIT1 |  |  | V |
| R4GNG3 | ARF GTPase-activating protein GIT2 (Fragment) |  | V |  |
| Q6N093 | Uncharacterized protein DKFZp686I04196 (Fragment) | V | V | V |
| A0A286YEY4 | Immunoglobulin heavy constant gamma 2 (Fragment) | V | V |  |
| A0A024RDY9 | Rho guanine nucleotide exchange factor (GEF) 7, isoform CRA_b | V | V | V |
| A0A804GS07 | Actin, cytoplasmic 2 | V |  |  |
| A0A0S2Z4Z9 | Non-POU domain containing octamer-binding isoform 1 (Fragment) | V | V | V |
| B3KPS3 | Tubulin alpha chain | V | V | V |
| A0A4D5RA86 | Annexin | V | V |  |
| Q5T0G8 | Annexin |  | V |  |
| A0A0S2Z2Z6 | Annexin ANXA6 |  | V | V |
| P08758  ANXA5 | Annexin A5 |  | V | V |
| A0A4D5RAB7 | Annexin |  |  | V |
| H0Y6E1 | Annexin A11; ANXA11 |  |  | V |
| A0A024R5Z7 | Annexin | V | V | V |
| B4DE59 | Junction plakoglobin | V | V | V |
| A0A024R882 | Stomatin, isoform CRA_a | V | V | V |
| Q8IWP6 | Tubulin beta chain | V | V | V |
| A0A087WUL4 | Retrotransposon-derived protein PEG10 | V | V | V |
| A0A286YEY1 | Immunoglobulin heavy constant alpha 1 (Fragment) | V | V | V |
| SWAHC | Ankyrin repeat domain-containing protein SOWAHC | V | V | V |
| V9HWA9 | Complement C3 | V | V | V |
| A2BDD9 | AMOT protein | V |  |  |
| Q4VCS5 | Angiomotin |  | V | V |
| B2R6J2  VIL2 | cDNA, FLJ92973, highly similar to Homo sapiens villin 2 (ezrin) (VIL2), mRNA | V | V | V |
| E9PPJ0 | Splicing factor 3B subunit 2 | V |  |  |
| Q2TA85 | C10orf54 protein (Fragment) | V | V | V |
| A0A1U9X7W7 | Epididymis secretory sperm binding protein | V |  |  |
| A0A384MDQ7 | Epididymis secretory sperm binding protein |  |  | V |
| Q2TSD0 | Glyceraldehyde-3-phosphate dehydrogenase | V | V | V |
| A0A024RDP4 | Paraspeckle component 1, isoform CRA_b | V | V | V |
| A8K6V3 | cDNA FLJ78677, highly similar to Homo sapiens splicing factor 3b, subunit 3, 130kDa (SF3B3), mRNA | V |  | V |
| SF3B3 | Splicing factor 3B subunit 3 |  | V |  |
| HS71A | Isoform 2 of Heat shock 70 kDa protein 1A | V | V | V |
| A0A0A0MQS7 | Estradiol 17-beta-dehydrogenase | V | V | V |
| Q0IIN1 | Keratin 77 | V | V |  |
| IGK | Immunoglobulin kappa light chain | V |  | V |
| A0A087WVQ9  Q9NZS6 | Elongation factor 1-alpha | V | V | V |
| DOCK7 | Isoform 2 of Dedicator of cytokinesis protein 7 | V | V | V |
| D3DSM4 | Collagen, type XVIII, alpha 1, isoform CRA_d | V | V | V |
| Q6NZ52 | 60S ribosomal protein L27a | V | V | V |
| Q8TBT6 | Uncharacterized protein (Fragment) | V | V | V |
| D2JYH4 | Actin, alpha 2, smooth muscle, aorta | V |  |  |
| ACE | Angiotensin-converting enzyme | V |  |  |
| ACE | Isoform Testis-specific of Angiotensin-converting enzyme P12821-3 |  | V | V |
| H0YEU2 | 40S ribosomal protein S3 (Fragment) | V |  |  |
| E9PL09 | 40S ribosomal protein S3 |  | V | V |
| A0A024R9C1 | Polyadenylate-binding protein | V | V | V |
| A0A024RAB6 | Heparan sulfate proteoglycan 2 (Perlecan), isoform CRA_b | V | V | V |
| Q6NZ55 | 60S ribosomal protein L13 | V | V | V |
| B2R491 | 40S ribosomal protein S4 | V | V | V |
| B5MCX3 | Septin-2 | V |  |  |
| TYY1 | Transcriptional repressor protein YY1 | V | V | V |
| B4DGF8 | cDNA FLJ57877, highly similar to Cleavage and polyadenylation specificity factor 7 | V | V | V |
| B9EG90 | DNA topoisomerase I | V | V | V |
| DPP9 | Isoform 2 of Dipeptidyl peptidase 9 | V | V | V |
| A0A024RD80 | Heat shock protein 90kDa alpha (Cytosolic), class B member 1, isoform CRA_a | V | V |  |
| A0A7P0T8Q5 | Transitional endoplasmic reticulum ATPase | V |  |  |
| A2A3R6 | 40S ribosomal protein S6 | V | V | V |
| A0A024R6I7 | Alpha-1-antitrypsin | V | V |  |
| B2RBU9 | cDNA, FLJ95704, highly similar to Homo sapiens serine protease inhibitor, Kunitz type 1 (SPINT1), mRNA | V |  |  |
| D3DP13 | Fibrinogen beta chain | V |  |  |
| V9HVY1 | Fibrinogen beta chain |  | V | V |
| A0A024R1K8 | Splicing factor 3a, subunit 1, 120kDa, isoform CRA_a | V | V | V |
| A1A508  PRSS3 | PRSS3 protein | V | V | V |
| A0RZB8 | Diaphanous-1, DIAPH1 | V | V |  |
| Q6NVC0 | ADP/ATP translocase (Fragment) | V | V | V |
| Q6I9V5 | ADP/ATP translocase (Fragment) |  |  | V |
| A0A0S2Z3H3 | ADP/ATP translocase (Fragment) |  |  | V |
| J3QQQ9 | KOW domain-containing protein | V | V | V |
| V9HW26 | ATP synthase subunit alpha | V | V | V |
| A0A7R6V2A8 | Intermediate-conductance Ca2+-activated K+ channel, KCa3.1 | V |  |  |
| H0Y362 | Zinc transporter (Fragment) | V |  | V |
| ZNT1 | Zinc transporter 1 | V | V | V |
| M0R0P7 | 60S ribosomal protein L18a | V |  |  |
| A0A1B0GTL5 | Rab11family-interacting protein 5 | V | V | V |
| Q86TT1 | Full-length cDNA clone CS0DD006YL02 of Neuroblastoma of Homo sapiens | V | V |  |
| A0A384MED8 | Epididymis secretory sperm binding protein | V | V | V |
| AHNK | Neuroblast differentiation-associated protein AHNAK | V | V | V |
| A0A7I2V2M5 | ATP-dependent RNA helicase DDX1 | V | V | V |
| E7EQ64 | Serine protease 1 | V |  |  |
| H0Y8D1 | Serine protease 1 (Fragment) |  | V | V |
| Q6FHP5  F5GY37 | Prohibitin | V | V | V |
| H7BY10 | 60S ribosomal protein L23a | V | V | V |
| A0A024QZD1 | Ribosomal protein L18, isoform CRA_c | V | V | V |
| P81605-2  DCD | Isoform 2 of Dermcidin | V |  | V |
| A0A5E4 | Uncharacterized protein | V | V | V |
| A0A6Q8PFK8 (HSPB1) | Heat shock protein beta-1 | V | V | V |
| Q6IPH7  A0PJ62 | 60S ribosomal protein L14 | V |  | V |
| FND3B  A0A024RDT9 | Fibronectin type III domain-containing protein 3B | V | V | V |
| A0A384NPU5 | Protein disulfide-isomerase A6 | V | V | V |
| A0A384MEJ3 | Epididymis secretory sperm binding protein | V | V | V |
| PRG2 | Isoform 2 of Bone marrow  proteoglycan | V | V | V |
| WDR5 | WD repeat-containing protein 5 | V |  |  |
| Q8N1G2  CMTR1 | Cap-specific mRNA (nucleoside-2'-O-)-methyltransferase 1 | V | V | V |
| A0A024R3A6 | Angiomotin like 1, isoform CRA_a | V | V | V |
| HIST1H4H (Q0VAS5; B2R4R0) | Histone H4 | V | V | V |
| A0A024R1X8 | Junction plakoglobin | V | V | V |
| HNRC4 | Heterogeneous nuclear ribonucleoprotein C-like 4 | V | V |  |
| Q86W19 | Protease serine 1 | V | V | V |
| A0A024R6W2 | Cleavage and polyadenylation specificity factor subunit 5; NUDT21 | V | V | V |
| B4DJI1 | L-lactate dehydrogenase | V | V | V |
| Q5U077 | L-lactate dehydrogenase |  | V |  |
| A0A5F9ZHM4 | L-lactate Dehydrogenase; LDHB |  |  | V |
| ZNT5  Q8TAD4 | Zinc transporter 5 | V | V | V |
| A0A7I2YQG2 | Ribosomal protein L19 | V | V | V |
| A0A7P0TAQ9 | DnaJ homolog subfamily C member 10 | V | V | V |
| H3BM89 | 60S ribosomal protein L4 | V |  |  |
| Q59GY2 | 60S ribosomal protein L4 (Fragment) |  | V | V |
| A0A0A6YYJ8 | Putative RNA-binding protein Luc7-like 2 | V |  | V |
| Q53SS8 | Epididymis secretory protein Li 85 | V | V | V |
| V9HWG7 | Epididymis secretory protein Li 52 |  | V |  |
| V9HWG3 | Epididymis secretory protein Li 45 |  |  | V |
| SMD2 | Isoform 2 of Small nuclear ribonucleoprotein Sm D2 | V |  |  |
| C9JNW5 | 60S ribosomal protein L24 | V | V | V |
| A0A075BPP5 | Methyl CpG binding protein 2 (Fragment) | V | V |  |
| A0A0A7M1X5 | Lamin B2, isoform CRA_b | V |  |  |
| A0A024RC55 | Milk fat globule-EGF factor 8 protein, isoform CRA_a | V | V | V |
| A0A024R5Z9 | Pyruvate kinase | V | V |  |
| V9HWB8 | Pyruvate kinase; HEL-S-30 |  |  | V |
| B2R984 | cDNA, FLJ94268, highly similar to Homo sapiens histone 1, H1e (HIST1H1E), mRNA | V |  |  |
| J3KQE5 | GTP-binding nuclear protein Ran (Fragment) | V | V | V |
| P80365  DHI2 | 11-beta-hydroxysteroid dehydrogenase type 2 | V | V | V |
| A0A8I5KUC3  D3DX01  Q49A63  MAOA | Amine oxidase; MAOA | V | V | V |
| A0A5C2GEJ8 | IGH c451_heavy__IGHV3-48_IGHD2-15_IGHJ6 (Fragment) | V |  |  |
| A0A2R8Y4T1 | Tensin-1 | V | V |  |
| G0XQ39 | STIM1L | V | V | V |
| E9PKZ0 | 60S ribosomal protein L8 (Fragment) | V | V | V |
| Q5JR94 | 40S ribosomal protein S8 | V | V | V |
| A0A0A0MRA8 | Band 4.1-like protein 3 | V | V | V |
| H0YEN5 | 40S ribosomal protein S2 (Fragment) | V | V | V |
| RELL1 | RELT-like protein 1 | V |  |  |
| S6AWF4 | IgG L chain | V |  |  |
| A0A0R7FJH5  F12 | Coagulation factor XII | V | V | V |
| A0A024RCX7 | SLC39A7 | V | V | V |
| A0A1S5UYZ9 | Diaphanous related formin 2 | V |  |  |
| H0Y8W2 | Receptor of-activated protein C kinase 1 (Fragment) | V | V |  |
| A0A0S2Z3R3 | Cholesterol side-chain cleavage enzyme, mitochondrial (Fragment) | V | V | V |
| NUDC1 | Isoform 2 of NudC domain-containing protein 1 | V | V | V |
| 3BHS1  P14060 | 3 beta-hydroxysteroid dehydrogenase/Delta 5-->4-isomerase type 1 | V | V | V |
| SC22B  O75396 | Vesicle-trafficking protein SEC22b | V | V | V |
| A0A8I5KR56 | Protein FAM98A | V | V |  |
| A8KA33 | cDNA FLJ76690, highly similar to Homo sapiens neuronal protein isoform a mRNA | V | V |  |
| A0A024R0C8 | Epididymis secretory sperm binding protein | V |  |  |
| A0A8I5KQ98 | Golgin subfamily A member 3 (Fragment) | V |  |  |
| A0A8I5KQH9 | Golgin subfamily A member 3 (Fragment) |  | V |  |
| A0A024R814 | Ribosomal protein L7, isoform CRA_a |  | V | V |
| A0A024R6I9 | Serpin peptidase inhibitor, clade A (Alpha-1 antiproteinase, antitrypsin), member 4,  isoform CRA_a | V | V | V |
| A0A024R7I3 | RAB8A, member RAS oncogene family | V |  |  |
| M0R0K9 | Transcription intermediary factor 1-beta | V |  |  |
| H0YKT5 | Transducin-like enhancer protein 3 | V | V |  |
| H0YKN8 | Transducin-like enhancer  protein 3 |  | V | V |
| HNRPK A0A024R228 | Heterogeneous nuclear ribonucleoprotein K | V | V | V |
| A0A024QZZ7 | Histone H2B; HIST1H2BD; HIST1H2BJ | V | V | V |
| A0A024R5S8 | Cytochrome P450, family 19, subfamily A, polypeptide 1, isoform CRA_a | V |  | V |
| A0A023T695 | Septin | V | V | V |
| A0A384N6H6 | Septin; SEPTIN2 |  | V | V |
| A0A087X142 | Septin; SEPTIN8 |  | V | V |
| Q96H79  ZCCHL | Zinc finger CCCH-type antiviral protein 1-like | V | V | V |
| B2R8I2 | cDNA, FLJ93914, highly similar to Homo sapiens histidine-rich glycoprotein (HRG), mRNA | V | V | V |
| A0A140VJS9 | Serine/threonine-protein phosphatase | V | V | V |
| A0A024R274 | Mothers against decapentaplegic homolog (SMAD4) | V | V | V |
| A0A096LNY5 | Adenylosuccinate lyase | V | V | V |
| A0A8I5KSZ3 | La-related protein 7 | V |  |  |
| A0A182DWH7 | Selenoprotein P (Fragment) | V | V | V |
| A0A024R5K8 | Serpin H1 | V | V | V |
| A0A024R0Y6 | ADP-ribosylation factor | V |  | V |
| H3BPJ9 | NADH dehydrogenase [ubiquinone] 1 beta subcomplex subunit 10; NDUFB10 | V | V | V |
| C4PGM0 | Specificity protein 1 | V |  |  |
| SMD3 | Isoform 2 of Small nuclear ribonucleoprotein Sm D3 | V |  |  |
| A0A5C2FTZ7 | IGL c202_light_IGKV3-20_IGKJ3 (Fragment) | V | V | V |
| KCRU | Isoform 2 of Creatine kinase U-type, mitochondrial | V |  | V |
| P12532  CKMT1B (KCRU) | Creatine kinase U-type, mitochondrial |  | V | V |
| A0A7I2V333 | Importin subunit alpha-3 | V |  |  |
| SEH1 | Isoform B of Nucleoporin SEH1 | V |  |  |
| A0A024RB87 | RAP1B, member of RAS oncogene family, isoform CRA_a | V |  | V |
| PITH1  Q9GZP4-2 | PITH domain-containing protein 1 | V | V | V |
| B4DIT7 | cDNA FLJ58187, highly similar to Protein-glutamine gamma-glutamyltransferase 2 | V | V |  |
| A0A024R845 | Ras-related protein Rab-14 | V | V | V |
| H0Y886 | NADH dehydrogenase [ubiquinone] 1 beta subcomplex subunit 5, mitochondrial | V | V |  |
| A0A024R4M0 | 40S ribosomal protein S9 | V |  | V |
| A0A384ME54 | Epididymis secretory sperm binding protein | V | V | V |
| B2RDQ3  SFRS10 | cDNA, FLJ96718, highly similar to Homo sapiens splicing factor, arginine/serine-rich 10 (transformer 2 homolog, Drosophila) (SFRS10), mRNA | V |  |  |
| B4DL99 | Dolichyl-diphosphooligosaccharide--protein glycosyltransferase subunit 1 | V |  |  |
| Q6IBR0 | Dolichyl-diphosphooligosaccharide--protein glycosyltransferase subunit 1 | V |  | V |
| A0A5C2FUF3 | IGL c382_light_IGKV3-20_IGKJ2 (Fragment) | V | V | V |
| A0A5C2FVH5 | IGL c909_light_IGKV4-1_IGKJ1 (Fragment) |  | V |  |
| A0A024QZE7 | Transforming growth factor beta 1 induced transcript 1 | V |  | V |
| A0A1B0GUN5 | Pleckstrin homology domain-containing family A member 6 | V | V | V |
| Q9Y3B4  SF3B6 | Splicing factor 3B subunit 6 | V | V | V |
| FABD  Q8IVS2 | Malonyl-CoA-acyl carrier protein transacylase, mitochondrial | V |  | V |
| A0A140VK14 | Glutamate dehydrogenase (NAD(P)(+)) | V |  |  |
| B4DMF5 | Glutamate dehydrogenase |  | V | V |
| A0A024R4F1 | Phosphopyruvate hydratase | V | V | V |
| A0A024R3X4 | 60 kDa heat shock protein, mitochondrial (Fragment) | V | V | V |
| B4DNU9 | cDNA FLJ55731, highly similar to Polymerase I and transcript release factor | V | V | V |
| A0A7P0NMY4 | Filamin-A | V |  |  |
| A0A1W2PP34 | Heterogeneous nuclear ribonucleoprotein U; HNRNPU | V |  | V |
| A0A1W2PP35 | Heterogeneous nuclear ribonucleoprotein U (Fragment) |  | V |  |
| B2RDY9 | Adenylyl cyclase-associated protein | V | V | V |
| A0A0D9SG04 | Cordon-bleu protein-like 1 | V |  | V |
| CATG | Cathepsin G | V |  | V |
| A0A068LL60 | Ig heavy chain variable region (Fragment) | V |  |  |
| A0A494C070 | Transcription factor Sp3 | V |  | V |
| LAMA5  O15230 | Laminin subunit alpha-5 | V | V | V |
| A0A7P0T892 | ADP-ribosylation factor-like protein 13B | V |  |  |
| V9HWP0 | Pentaxin | V | V | V |
| SP6 | Transcription factor Sp6 | V |  |  |
| A0A024R1E2 | Myosin XVIIIB, isoform CRA_c | V |  |  |
| C4A | Isoform 2 of Complement C4-A | V | V |  |
| A0A024R866 | 60S ribosomal protein L35 | V | V | V |
| A1LUY1  RPL34 | 60S ribosomal protein L34 | V | V | V |
| Q5T8U2 | 60S ribosomal protein L7a | V |  |  |
| A0A2R8Y811 | 40S ribosomal protein S14 (Fragment) | V |  | V |
| Q5U0A5  RAD51 | DNA repair protein RAD51 homolog | V | V | V |
| A0A0M4FNU3 | Fructose-bisphosphate aldolase | V |  |  |
| M0R2N5 | Very-long-chain enoyl-CoA reductase; TECR | V | V |  |
| BI2L1 | Brain-specific angiogenesis inhibitor 1-associated protein 2-like protein 1 | V | V | V |
| A0A1C7CYX9 | Dihydropyrimidinase-related protein 2 (DPYSL2) | V | V | V |
| A8K517 | 40S ribosomal protein S23 | V | V | V |
| ROA0  Q13151 | Heterogeneous nuclear ribonucleoprotein A0; HNRNPA0 | V |  | V |
| A0A0K0K1K4 | Proteasome subunit alpha type | V | V | V |
| TGVO1 | Isoform 2 of Transport and Golgi organization protein 1 homolog | V |  |  |
| VXP32  Q5T750 | Skin-specific protein 32 | V | V |  |
| A0A248RGE3 | Ubiquitin-40S ribosomal protein S27a | V | V | V |
| M0R1H5 | 40S ribosomal protein S11 | V |  |  |
| M0QZC5 (RPS11) | 40S ribosomal protein S11 |  | V | V |
| H0YH81 | ATP synthase subunit beta | V |  |  |
| Q0QEN7  ATP5B | ATP synthase subunit beta (Fragment) |  | V | V |
| A0A024RA55 | Glycoprotein (Transmembrane) nmb, isoform CRA_b | V |  |  |
| M0R0F9 | Cdc42-interacting protein 4 | V |  |  |
| B3KXN4 | cDNA FLJ45763 fis, clone N1ESE2000698, highly similar to WD repeat protein 1 | V |  | V |
| A0A024RAD5 | Dolichyl-diphosphooligosaccharide--protein glycosyltransferase 48 kDa subunit | V | V | V |
| A0A0U1RQX8 | E3 ubiquitin-protein ligase CBL | V | V | V |
| A0A8I5KNU9 | Protein disulfide-isomerase | V | V |  |
| B3KQT9 | Protein disulfide-isomerase |  |  | V |
| B2R6C4 | Receptor expression-enhancing protein | V |  |  |
| F6KPG5 | Albumin (fragment) |  | V |  |
| FLNA  P21333-2 | Isoform 2 of Filamin-A |  | V | V |
| KTN1 | Isoform 2 of Kinectin |  | V | V |
| A0A0S2Z3G9 | Actinin alpha 4 isoform 1 (Fragment) |  | V | V |
| A0A024R694 | Actinin, alpha 1 |  |  | V |
| A0A0S2Z428 | HCG2039812, isoform CRA_b (Fragment) |  | V | V |
| K2C6B | Keratin, type II cytoskeletal 6B |  | V | V |
| A0A1S5UZ07 | Talin-1 |  | V |  |
| A0A024R321 | Filamin B, beta (Actin binding protein 278), isoform CRA_a |  | V | V |
| Q1KLZ0 | HCG15971, isoform CRA_a |  | V | V |
| DESP  P15924 | Desmoplakin |  | V | V |
| Q6GMX6 | IGH@ protein |  | V | V |
| A8K008 | Uncharacterized protein |  | V | V |
| A0A024R6B5 | Heat shock 70kDa protein 2, isoform CRA_a |  | V | V |
| A8K3K1 | cDNA FLJ78096, highly similar to Homo sapiens actin, alpha, cardiac muscle (ACTC), mRNA |  | V | V |
| Q6N094 | Uncharacterized protein DKFZp686O01196 |  | V |  |
| Q86YZ3  HORN | Hornerin |  | V | V |
| G3V0E5  A8K6Q8 | Transferrin receptor protein 1 |  | V | V |
| Q6N095 | Uncharacterized protein |  | V | V |
| Q4TWB7 | Beta globin |  | V |  |
| Q2VPJ6 | HSP90AA1 protein (Fragment) |  | V | V |
| A0A7P0TAW3 | Transitional endoplasmic reticulum ATPase |  | V | V |
| K1C17 | Keratin, type I cytoskeletal 17 |  | V |  |
| A0A1K0GXZ1 | Globin C1 |  | V |  |
| Q6PIL8 | IGK@ protein |  | V | V |
| Q6P5S8 | IGK@ protein |  |  | V |
| FMNL2 | Formin-like protein 2 |  | V | V |
| SF3B2 | Splicing factor 3B subunit 2 |  | V | V |
| A0A1X7SBZ2 | RNA helicase |  | V | V |
| A2KBC2 | Anti-(ED-B) scFV (Fragment) |  | V | V |
| Q6N030 | Uncharacterized protein |  | V | V |
| DSG1  Q02413 | Desmoglein-1 |  | V | V |
| RTCB | RNA-splicing ligase RtcB homolog |  | V |  |
| A7BI36 | p180/ribosome receptor |  | V | V |
| B7ZLJ0 | DIAPH2 protein |  | V |  |
| SPIT1 | Isoform 2 of Kunitz-type protease inhibitor 1 |  | V | V |
| A0A087WVQ6 | Clathrin heavy chain |  | V | V |
| A0A2X0SFD1 | DOCK8 (Fragment) |  | V |  |
| COL14A1 | Isoform 2 of Collagen alpha-1(XIV) chain |  | V | V |
| V9HWA5 | Calponin |  | V |  |
| D1MQ09 | Small conductance potassium channel type4 transcript variant 3 |  | V |  |
| A0A024RC65 | HCG1991735, isoform CRA_a |  | V | V |
| V9HWC0 | Epididymis luminal protein 70 |  | V | V |
| A3R0T7 | Liver histone H1e |  | V | V |
| ZNT7 | Zinc transporter 7 |  | V | V |
| KRT72 | Isoform 2 of Keratin, type II cytoskeletal 72 |  | V |  |
| A0A7N4I394 | Pre-mRNA-processing factor 40 homolog A |  | V | V |
| KRT78  Q8N1N4 | Keratin, type II cytoskeletal 78 |  | V | V |
| C9J8T0 | Selenocysteine-specific elongation factor |  | V |  |
| A0A024R3E3 | Apolipoprotein A-I, isoform CRA_a |  | V | V |
| Q53GL5 | Isocitrate dehydrogenase (NADP(+)) (Fragment) |  | V | V |
| D9ZGG2 | Vitronectin |  | V | V |
| B0YJC4 | Vimentin |  | V | V |
| A0A024R6C9 | Dihydrolipoyllysine-residue succinyltransferase component of  2-oxoglutarate dehydrogenase complex, mitochondrial |  | V | V |
| FN1 (A0A024R462) | Fibronectin |  | V | V |
| RPS9 (A5D904) | RPS9 protein |  | V |  |
| PPIB (A0A7P0TB45) | Peptidyl-prolyl cis-trans isomerase |  | V |  |
| A0A024QZN4 | Vinculin |  | V | V |
| A0A7S5EXU8 | IGH c2819_heavy_IGHV3-30_IGHD3-16_IGHJ4 (Fragment) |  | V |  |
| D3DWB6 | Ubiquitinyl hydrolase 1 |  | V | V |
| B2RDK6 (SPFH1) | cDNA, FLJ96656, highly similar  to Homo sapiens SPFH domain family, member 1 (SPFH1), mRNA |  | V |  |
| B2R6T2 | cDNA, FLJ93100, highly similar  to Homo sapiens calpain 6 (CAPN6), mRNA |  | V | V |
| A0A140VKE1 | UTP--glucose-1-phosphate ridylyltransferase |  | V | V |
| A0A0A0MSI0 | Peroxiredoxin-1 (Fragment) |  | V | V |
| A0A024RBS2 | 60S acidic ribosomal protein P0 |  | V | V |
| B4DRR7 | cDNA FLJ59399, highly similar  to Keratin, type II cytoskeletal 4 |  |  |  |
| A0A384P5Q0 | Catalase |  | V | V |
| B4E3M6 | cDNA FLJ55446, highly similar  to Superkiller viralicidic activity 2-like 2 |  | V | V |
| A0A5C2GG23 | IG c630_heavy_IGHV4-39_IGHD6-19_IGHJ4 (Fragment) |  | V |  |
| Q6FIG4 (RAB1B) | RAB1B protein |  | V | V |
| Q5U0I6 | H.sapiens ras-related Hrab1A  protein |  | V | V |
| SR140 | Isoform 2 of U2 snRNP-associated SURP motif-containing protein U2SURP |  | V | V |
| UBASH3B | Ubiquitin-associated and SH3  domain-containing protein B |  | V |  |
| CKMT1B | Creatine kinase U-type, mitochondrial |  | V |  |
| P02790; HPX | Hemopexin |  | V | V |
| GANAB (A0A024R592) | Glucosidase, alpha neutral AB, isoform CRA_b |  | V |  |
| A0A024R9Y7 | Melanoma antigen family D, 2 |  | V | V |
| H0YKD8 | 60S ribosomal protein L28 |  | V | V |
| ITGA6 A0A8C8KBL6 | Integrin alpha-6 |  | V | V |
| A0A024RA24 | zinc_ribbon_16 domain-containing protein , GATOR complex protein MIOS , FLJ20323 |  | V | V |
| M0R2H7 | Cdc42-interacting protein 4 |  | V | V |
| B2R8R5 | cDNA, FLJ94025, highly similar to Homo sapiens tripartite motif-containing 28 (TRIM28), mRNA |  | V |  |
| B4DST7 | cDNA FLJ58406, weakly similar  to Mus musculus experimental autoimmune prostatitis antigen 2 (Eapa2) mRNA |  | V | V |
| A0A087WYK1 | Pregnancy-specific beta-1-glycoprotein 9, PSG9 |  | V | V |
| B2RE46 | Dolichyl-diphosphooligosaccharide--protein glycosyltransferase subunit 2 |  | V | V |
| Q6KB66-3 | Isoform 3 of Keratin, type II cytoskeletal 80, KRT80 |  | V |  |
| A0A7P0T937  CANX | Calnexin, CANX |  | V | V |
| B2R6V9 | cDNA, FLJ93141, highly similar to Homo sapiens coagulation factor XIII, A1 polypeptide (F13A1), mRNA |  | V | V |
| P02671-2 | Isoform 2 of Fibrinogen alpha chain |  | V | V |
| A0A024R2Q3 | Catenin (Cadherin-associated protein), beta 1, 88kDa, isoform CRA_a, CTNNB1 |  | V | V |
| A0A024R1V4 | 60S ribosomal protein L27 |  | V | V |
| C9J2Z4  A0A024R571 | EH domain-containing protein 1 (Fragment), EHD1 |  | V | V |
| P35030 | Trypsin-3, PRSS3 |  | V |  |
| A0A140VKA0 | Caldesmon 1, isoform CRA_i, CALD1 |  | V | V |
| B4DUI5  TPI1 | Triosephosphate isomerase |  | V | V |
| A0A384ME17 | Elongation factor Tu |  | V | V |
| A0A090N8Z4 | Immunity associated protein 1, IMAP1 |  | V |  |
| A0A024RDB4 | Heterogeneous nuclear ribonucleoprotein D (AU-rich element RNA binding protein 1, 37kDa), isoform CRA_c; HNRPD |  | V | V |
| A0A2X0TVX1 | DOCK6 (Fragment) |  | V | V |
| A0A024R261 | 60S ribosomal protein L17, hCG_24487 |  | V | V |
| P30050 | 60S ribosomal protein L12 |  | V | V |
| Q05CU5 | RASA1 protein (Fragment); RASA1 |  | V |  |
| A0A7I2V2R5  DPP4 | Dipeptidyl peptidase 4; DPP4 |  | V | V |
| A0A0B4J259 | Lysozyme C |  | V | V |
| P62269 | 40S ribosomal protein S18 |  | V | V |
| C1K3N0  A0A6E1XE14 | Transcription factor AP-2-alpha; TFAP2A |  | V | V |
| H0YD14 | Myoferlin (Fragment) |  | V | V |
| O60885 | Bromodomain-containing protein 4; BRD4 |  | V | V |
| A0A024R962 | HCG40889, isoform CRA_b |  | V | V |
| Q7Z6R9 | Transcription factor AP-2-delta |  | V |  |
| A0A024R324 | Epididymis secretory sperm binding protein; RHOA |  | V | V |
| A0A384NPH0 | Epididymis secretory sperm binding protein |  | V | V |
| Q1L857 | Ceruloplasmin (Fragment) |  | V | V |
| A0A024R1S8 | LIM and SH3 domain protein 1 |  | V | V |
| P78527-2 | Isoform 2 of DNA-dependent protein kinase catalytic subunit; PRKDC |  | V | V |
| A0A5C2G716 | IGH c427_heavy__IGHV3-74_IGHD7-27_IGHJ4 (Fragment) |  | V |  |
| B3KPA6 | Acyl-Coenzyme A dehydrogenase, very long chain, isoform CRA_e; ACADVL |  | V | V |
| A0A024RA28 | Heterogeneous nuclear ribonucleoprotein A2/B1, isoform CRA_d; HNRNPA2B1 |  | V |  |
| Q7Z759 | T-complex protein 1 subunit theta; CCT8 |  | V | V |
| B3KVN0 | Solute carrier family 2, facilitated glucose transporter member 1 |  | V | V |
| K7EJT5 | 60S ribosomal protein L22 (Fragment); RPL22 |  | V | V |
| P62851 | 40S ribosomal protein S25; RPS25 |  | V | V |
| P55268 | Laminin subunit beta-2; LAMB2 |  | V | V |
| F8WJN3 | Cleavage and polyadenylation specificity factor subunit 6; CPSF6 |  | V | V |
| A0A3B3IRQ7 | Alpha-2-macroglobulin receptor-associated protein; LRPAP1 |  | V |  |
| A0A590UJL0 | Steryl-sulfatase; STS |  | V |  |
| A0A024RBK3 | 60S ribosomal protein L6 |  | V | V |
| A0A1B0GVD3 | Protein lin-28 homolog B; LIN28B |  | V | V |
| A0A0C4DGH5 | Cullin-associated NEDD8-dissociated protein 1 (Fragment); CAND1 |  | V |  |
| A0A6I8PRN4 | Glutamine--fructose-6-phosphate transaminase (isomerizing) |  | V | V |
| A0A2R8Y443 | Dual-specificity kinase (Fragment); DYRK1A |  | V | V |
| A0A384P5V3 | Epididymis secretory sperm binding protein |  | V | V |
| P07814 | Bifunctional glutamate/proline--tRNA ligase |  | V | V |
| E9PBF6 | Lamin-B1 |  | V | V |
| B3KU87 | Solute carrier family 30 (Zinc transporter), member 6, isoform CRA_c |  | V | V |
| Q15717-2  ELAV1 | Isoform 2 of ELAV-like protein 1; ELAVL1 |  | V | V |
| P25815 | Protein S100-P; S100P |  | V | V |
| B2R5H0 | Protein S100 |  | V | V |
| A0A024R9D3 | 60S ribosomal protein L30 |  | V | V |
| B4DDF7 | cDNA FLJ53296, highly similar to Serine/threonine-protein phosphatase 2A 65 kDa regulatory subunit A alpha isoform |  | V |  |
| Q99988 | Growth/differentiation factor 15; GDF15 |  | V | V |
| Q5D862 | Filaggrin-2 |  | V | V |
| A0A090N8Y2 | Protein disulfide-isomerase A4 |  | V | V |
| D3DTX7 | Collagen, type I, alpha 1, isoform CRA_a |  | V |  |
| A0A7I2V5T6  Q8TAG3  SLC4A2 | Anion exchange protein; SLC4A2 |  | V | V |
| A0A087WU93 | AP-2 complex subunit beta (Fragment); AP2B1 |  | V | V |
| P05111 | Inhibin alpha chain |  | V |  |
| B4DVM5 | GATOR complex protein WDR24 |  | V | V |
| Q5JQF8 | Polyadenylate-binding protein 1-like 2 |  | V |  |
| B7Z2F6 | cDNA FLJ54744, highly similar to Scaffold attachment factor B |  | V |  |
| B4DEA3 | UV excision repair protein RAD23 |  | V | V |
| Q6FH10 | Decorin |  | V |  |
| Q5TEZ5 | Uncharacterized protein C6orf163 |  | V |  |
| H7C0R7 | Cytochrome-b5 reductase (Fragment) |  | V | V |
| H0YA05 | Palladin |  | V |  |
| A0A384P5G6 | Stress-70 protein, mitochondrial |  | V | V |
| A0A7S5C1Z3 | IGH c2621_heavy_IGHV4-39_IGHD3-10_IGHJ6 (Fragment) |  | V |  |
| A0A024R5S8 | Cytochrome P450, family 19, subfamily A, polypeptide 1, isoform CRA_a |  | V |  |
| D6W539  HADHB | Hydroxyacyl-Coenzyme A dehydrogenase/3-ketoacyl-Coenzyme A thiolase/enoyl-Coenzyme A hydratase (Trifunctional protein), beta subunit, isoform CRA_b |  | V |  |
| X1WI28 | 60S ribosomal protein L10 (Fragment) |  | V | V |
| E9PQU5  P49756 | RNA-binding protein 25 (Fragment) |  | V | V |
| A0A024R566 | Splicing factor 1 |  | V | V |
| B7Z881 | cDNA FLJ52376, highly similar to Ras GTPase-activating-like protein IQGAP2 |  | V |  |
| A0A024RAJ8  IQGAP2 | IQ motif containing  GTPase activating protein 2, isoform CRA_b |  |  | V |
| H0Y8L7 | 40S ribosomal protein S3a (Fragment) |  | V |  |
| F5H563 | Protein 4.2 |  | V |  |
| A0A384ME37 | 60S ribosomal protein L13a |  | V | V |
| A0A024R5K1 | Coronin |  | V |  |
| A0A024R1N4 | X-ray repair complementing defective repair in Chinese hamster cells 6 (Ku autoantigen, 70kDa) |  |  | V |
| A0A024R872 | Chromosome 9 open reading frame 88 |  | V | V |
| A0A3B3IU24 | Serine protease HTRA1 |  | V |  |
| A0A7P0TA23 | O-phosphoseryl-tRNA(Sec) selenium transferase; SEPSECS |  | V | V |
| A0A384MR50 | F-actin-capping protein subunit beta |  | V | V |
| Q7L2M7 | PFKL protein (Fragment) |  | V | V |
| Q53YD7 | Elongation factor 1-gamma |  | V | V |
| A0A384MTR5 | RuvB-like helicase |  | V | V |
| H0YFA4 | Cysteine-rich protein 2 (Fragment); CRIP2 |  | V | V |
| Q65ZQ3 | FBRNP; D10S102 |  | V |  |
| A0A7I2V5R6 | Guanine nucleotide-binding protein G(s) subunit alpha isoforms short |  | V | V |
| A0A7I2V620 | NADH dehydrogenase [ubiquinone] 1 beta subcomplex subunit 9 |  | V |  |
| A0A087WZX2 | NADH dehydrogenase [ubiquinone] 1 beta subcomplex subunit 6 |  | V |  |
| H7C0A3 | ARPC4-TTLL3 readthrough (Fragment); GOSR2 |  | V | V |
| A0A024RBM3 | Ubiquitin carboxyl-terminal hydrolase |  | V | V |
| Q8TCJ2 | Dolichyl-diphosphooligosaccharide--protein glycosyltransferase subunit STT3B |  | V | V |
| Q5QPL9 | RNA-binding protein Raly |  | V |  |
| Q05DF2 | SF3A2 protein; SF3A2 | V | V | V |
| A4D1P0 | Aldo_ket_red domain-containing protein |  | V | V |
| Q9BXP8-2 | Isoform 2 of Pappalysin-2; PAPPA2 |  | V | V |
| A0A140VK44 | Proteasome subunit alpha type |  | V |  |
| Q9Y3U8 | 60S ribosomal protein L36 |  | V | V |
| A0A024RCM3 | RNA helicase |  | V |  |
| A0A590UJ86  DLG1 | Disks large homolog 1 |  | V | V |
| A0A024R1K7 | Tyrosine 3-monooxygenase/tryptophan 5-monooxygenase activation protein, eta  polypeptide |  | V | V |
| A0A024RAL1 | Chondroitin sulfate  proteoglycan 2 (Versican), isoform CRA_c |  | V | V |
| A0A024R0S6 | EH-domain containing 2, isoform CRA_a |  | V | V |
| A0A024RAI1 | Actin-related protein 3 |  | V | V |
| Q5HYC5 | Uncharacterized protein  DKFZp686L08115 (Fragment) |  | V |  |
| B3KSR8 | cDNA FLJ36832 fis, clone  ASTRO2010799, highly similar to ALPHA-1 CATENIN |  | V |  |
| H0YB24 | Cell cycle and apoptosis  regulator protein 2 (Fragment); CCAR2 |  | V |  |
| Q96ST3 | Paired amphipathic helix protein Sin3a |  | V | V |
| A0A140TA62 | IF rod domain-containing protein |  | V |  |
| A0A024QZ91  ATXN2L | Ataxin 2-like |  | V | V |
| F8WB05 | Ataxin-2; ATXN2 |  |  | V |
| A0A3B3ITK7 | Phosphoglucomutase-1; PGM1 |  | V | V |
| B3KWV6 | Cytoplasmic FMR1-interacting protein |  | V |  |
| A0A494C0H6 | Transcriptional activator protein Pur-alpha |  | V |  |
| A4D2P0 | Ras-related C3 botulinum toxin substrate 1 (Rho family, small GTP binding protein Rac1); RAC1 |  | V |  |
| B0QY89 | Eukaryotic translation initiation factor 3 subunit L |  | V | V |
| B4DK16 | cDNA FLJ57882, highly similar  to Pre-mRNA-processing-splicing factor 8 |  | V |  |
| A0A0A0MS51 | Gelsolin |  | V | V |
| A0A024RAA5  CDC42 | Cell division control protein 42 homolog; CDC42 |  | V | V |
| A0A024R413 | NADH dehydrogenase [ubiquinone] 1 beta subcomplex subunit 3; NDUFB3 |  | V | V |
| A0A2X0SF71  ARHGAP17 | ARHGAP17 (Fragment); ARHGAP17 |  | V |  |
| A0A7I2V2F9  ITGB1 | Integrin beta |  | V | V |
| O60763-2  USO1 | Isoform 2 of General vesicular transport factor p115 |  | V |  |
| A0A494C0J7 | TGc domain-containing protein |  | V |  |
| B2RCU6 | Serine/threonine-protein kinase PAK 3 |  | V |  |
| A0A0C4DGV8 | Semaphorin-3B |  | V | V |
| Q14554  PDIA5 | Protein disulfide-isomerase A5 |  | V |  |
| A0A075B6E2 | 40S ribosomal protein S19 |  | V | V |
| A0A7P0T8D1  AGT | Angiotensinogen |  | V | V |
| E7DVW5 | Fatty acid binding protein 5 (Psoriasis-associated); FABP5 |  | V |  |
| Q7L2E3-2  DHX30 | Isoform 2 of ATP-dependent RNA helicase DHX30 |  | V | V |
| B4DEQ7 | cDNA FLJ50003, highly similar to Homo sapiens leucine-rich repeats and calponin homology (CH) domain containing 3 (LRCH3), mRNA |  | V |  |
| C1PHA2 | Tyrosine-protein kinase receptor |  | V | V |
| Q9HB00 | Desmocollin 1, isoform CRA_b |  | V | V |
| Q7L2J0 | 7SK snRNA methylphosphate capping enzyme |  | V | V |
| Q5T749 | Keratinocyte proline-rich Protein; KPRP |  | V |  |
| A0A3B3IS84 | Coatomer subunit alpha |  | V | V |
| A0A7I2V4E8  TRPM3 | Transient receptor potential cation channel subfamily M member 3 |  | V |  |
| A0A2X0SSH0 | ARHGEF6 (Fragment) |  | V |  |
| B4DM22 | 26S proteasome non-ATPase regulatory subunit 2 |  | V | V |
| A0A024R702 | Tubulin polymerization-promoting protein family member 3 |  | V | V |
| A0A5C2FSY5 | IGL c28_light_IGKV1-5_IGKJ1 (Fragment) |  | V |  |
| A0A0E3XJU3 | Cadherin-1 |  | V |  |
| A0A024RB16  FAM62A | Family with sequence similarity 62 (C2 domain containing), member A; FAM62A |  | V |  |
| A0A024RBB5 | Cysteine and glycine-rich protein 2, isoform CRA_a; CSRP2 |  | V | V |
| A0A0S2Z3H5 | Collagen type I alpha 2 isoform 1 (Fragment); COL1A2 |  | V |  |
| P21128-2  ENDOU | Isoform 2 of Uridylate-specific endoribonuclease |  | V | V |
| A0A024R6N9 | Serpin peptidase inhibitor, clade A (Alpha-1 antiproteinase, antitrypsin), member 5; SERPINA5 |  | V | V |
| DOCK4  A0A2X0SFR8 | DOCK4; Dedicator of cytokinesis protein 4 |  | V |  |
| PLRG1 | Isoform 2 of Pleiotropic regulator 1 |  | V | V |
| A8KAK1 | cDNA FLJ77398, highly similar to Homo sapiens UDP-glucose ceramide glucosyltransferase-like 1, transcript variant 2 |  | V |  |
| A0A2Q3DQE3 | Calcium/calmodulin-dependent protein kinase; CAMK2G |  | V | V |
| H0YAY3 | DENN domain-containing protein 3 |  | V |  |
| A0A087WV90 | Dystrophin |  | V |  |
| Q96A22 | Uncharacterized protein C11orf52 |  | V | V |
| A0A140VK46 | Proteasome subunit beta |  | V | V |
| A0A024RDH6 | SEC31-like 1 |  | V | V |
| LLGL2  A0PJJ0 | LLGL2 protein |  | V |  |
| RNF40  A0A0S2Z537 | E3 ubiquitin protein ligase |  | V |  |
| A0A0B4J1Z1 | Serine/arginine-rich-splicing factor 7 |  | V |  |
| Q3SY56 | Transcription factor Sp6 |  | V |  |
| A0A024R972  LAMC1 | Laminin, gamma 1 (Formerly LAMB2) |  | V | V |
| B3KS36 | cDNA FLJ35376 fis, clone SKMUS2004044, highly similar to Homo sapiens ribosomal protein L3 (RPL3), transcript variant 2 |  | V | V |
| Q92522 | Histone H1.10 |  | V | V |
| A0A087X208 | Agrin; AGRN |  | V |  |
| F8W930 | Insulin-like growth factor 2 mRNA-binding protein 2 |  | V | V |
| A0A2R8Y5Y7 | 60S ribosomal protein L9 |  | V |  |
| Q9HB75-2  PIDD1 | Isoform 2 of p53-induced death  domain-containing protein 1 |  | V |  |
| A0A0D9SEI3 | Cyclin-dependent kinase 11B |  | V | V |
| B3KTN5 | cDNA FLJ38538 fis, clone HCHON2001407, highly similar to LanC-like protein 2 |  | V | V |
| P07199 | Major centromere autoantigen B; CENPB |  | V | V |
| A0A024R8Q0  SEPT9 | Septin 9 |  | V |  |
| A0A0S2Z3L4  CTSD | Cathepsin D isoform 2; CTSD |  | V |  |
| F2Z2C7 | Protein transport protein Sec61 subunit alpha isoform 2 |  | V |  |
| A0A024R4Q8 | Ribosomal protein S5 |  | V |  |
| A0A0C4DG40 | Nesprin-1 |  | V |  |
| A0A024RCT9  HMGA1 | High mobility group  AT-hook 1 |  | V |  |
| A0A090N7V5 | Gamma-glutamylcyclotransferase |  | V |  |
| S4R3B5 | Protein transport protein Sec61 subunit beta |  | V |  |
| H0YCU9 | Transgelin |  | V |  |
| H0YJV3 | Nidogen-2 |  | V | V |
| Q9Y4H2 | Insulin receptor substrate 2; IRS2 |  | V | V |
| A0A0A0MT26 | Sodium/potassium-transporting ATPase subunit alpha-3; ATP1A3 |  | V | V |
| A0A024R9A4 | RNA helicase; DDX3Y |  | V | V |
| A0A0G2JLD8  SSBP1 | Single-stranded DNA-binding protein, mitochondrial |  | V | V |
| B3KPR6 | cDNA FLJ32099 fis, clone OCBBF2001140, weakly similar to VACUOLAR PROTEIN SORTING-ASSOCIATED PROTEIN VPS8 |  | V |  |
| B3KR56 | cDNA FLJ33721 fis, clone BRAWH2016792, highly similar to Angiomotin-like protein 2 |  | V |  |
| F5GX11 | Proteasome subunit alpha type-1 |  | V | V |
| A0A024R824 | Copper transport Protein; SLC31A1 |  | V | V |
| B4DHB3  PGK2; PGK1 | Phosphoglycerate kinase |  | V | V |
| A0A075B716 | 0S ribosomal protein S17 |  | V | V |
| A0A2R8Y5M6 | B-cell receptor-associated protein |  | V |  |
| B7Z741 | cDNA FLJ50712, highly similar to Zinc finger protein 406 |  | V |  |
| K7EJB5  SNRPD2 | Small nuclear ribonucleoprotein Sm D2 |  | V | V |
| A0A087WU53 | Magnesium transporter protein 1 |  | V | V |
| B4DPF1 | cDNA FLJ56358, highly similar to Cytosolic nonspecific dipeptidase |  | V | V |
| A0A024QZN9  VDAC2 | Voltage-dependent  anion-selective channel protein 2; VDAC2 |  | V | V |
| J3KMX5 | 40S ribosomal protein S13 |  | V |  |
| A0A2R8Y595 | Transcriptional repressor CTCF |  | V | V |
| H3BMH3 | GATOR complex protein WDR59 |  | V | V |
| Q7Z417 | FMR1-interacting protein NUFIP2 |  | V | V |
| KNL1  Q8NG31-2 | Isoform 2 of Kinetochore scaffold 1 |  | V | V |
| A0A087X2I1 | 26S proteasome regulatory subunit 10B; PSMC6 |  | V |  |
| B4DYR6 | Dynamin-1-like protein |  | V |  |
| A0A024QYU9 | Eukaryotic translation initiation factor 3 subunit C |  | V |  |
| A0A087WW66 | 26S proteasome non-ATPase regulatory subunit 1 |  | V |  |
| A0A0D9SEJ5 | Constitutive coactivator of peroxisome proliferator-activated receptor gamma; FAM120B |  | V | V |
| A0A024RBE8 | Phosphate carrier protein, mitochondrial |  | V | V |
| P35908 | Keratin, type II  cytoskeletal 2 epidermal ; K22E |  |  | V |
| P13645 | Keratin, type I cytoskeletal 10 |  |  | V |
| A0A8I5KSF0  GOLGA3 | Golgin subfamily A member 3 |  |  | V |
| Q9Y490 | Talin-1 |  |  | V |
| A0A140VKC4 | Methyl-CpG-binding protein 2; MECP2 |  |  | V |
| O60610 | Protein diaphanous homolog 1; DIAPH1 |  |  | V |
| E9PCR7 | Oxoglutarate dehydrogenase (succinyl-transferring) |  |  | V |
| B4DW52 | cDNA FLJ55253, highly similar to Actin, cytoplasmic 1 |  |  | V |
| B4DMA2 | cDNA FLJ54023, highly similar to Heat shock protein HSP 90-beta |  |  | V |
| Q13263 | Transcription intermediary factor 1-beta; TRIM28 |  |  | V |
| B2R853 | cDNA, FLJ93744, highly similar to Homo sapiens keratin 6E (KRT6E), mRNA |  |  | V |
| B2R950 | cDNA, FLJ94213, highly similar to Homo sapiens pregnancy-zone protein (PZP), mRNA |  |  | V |
| P0DOX5 | Immunoglobulin gamma-1 heavy chain |  |  | V |
| V9HW68 | Epididymis luminal protein 214 |  |  | V |
| A8K5F7 | cDNA FLJ75405, highly similar to Homo sapiens diaphanous homolog 2 (Drosophila) (DIAPH2), transcript variant 12C, mRNA |  |  | V |
| A0A140VJJ6 | Testicular tissue protein Li 70 |  |  | V |
| Q14161-5  GIT2 | Isoform 5 of ARF  GTPase-activating protein GIT2 |  |  | V |
| Q6N092 | Uncharacterized protein DKFZp686K18196 |  |  | V |
| Q9Y2L9-2  LRCH1 | Isoform 2 of Leucine-rich repeat and calponin homology domain-containing protein 1 |  |  | V |
| Q9Y3I0 | RNA-splicing ligase RtcB homolog |  |  | V |
| Q49A63  MAOA | Amine oxidase |  |  | V |
| P13010 | X-ray repair cross-complementing protein 5 |  |  | V |
| P62424 | 60S ribosomal protein L7a |  |  | V |
| P01871-2  IGHM | Isoform 2 of Immunoglobulin heavy constant mu |  |  | V |
| B4DJ30 | cDNA FLJ61290, highly similar to Neutral alpha-glucosidase AB |  |  | V |
| E9KL35 | Epididymis tissue sperm binding protein Li 3a |  |  | V |
| B4DTL8 | cDNA FLJ61389, highly similar to Ras GTPase-activating protein 1 |  |  | V |
| Q5JRP2 | Disintegrin and metalloproteinase domain-containing protein 12; ADAM12 |  |  | V |
| A0A0C4DGL8 | Haptoglobin |  |  | V |
| A2A369 | Dedicator of cytokinesis protein 8; DOCK8 |  |  | V |
| A8K8U1 | cDNA FLJ77762, highly similar to Homo sapiens cullin-associated and neddylation-dissociated 1 (CAND1), mRNA |  |  | V |
| P57772 | Selenocysteine-specific elongation factor |  |  | V |
| A0A286YFJ8 | Immunoglobulin heavy constant gamma 4 (Fragment); IGHG4 |  |  | V |
| O43776 | Asparagine--tRNA ligase, cytoplasmic |  |  | V |
| A0A2R8Y5S7 | Radixin |  |  | V |
| P0C0S8 | Histone H2A type 1; H2AC17 |  |  | V |
| A0A0U1RR32 | Histone H2A |  |  | V |
| O75367-2  MACROH2A1 | Isoform 1 of Core histone macro-H2A.1; MACROH2A1 |  |  | V |
| Q8TF42 | Ubiquitin-associated and SH3 domain-containing protein B; UBASH3B |  |  | V |
| Q14151 | Scaffold attachment factor B2 |  |  | V |
| A0A7I2V340  KPNA4 | Importin subunit alpha; KPNA4 |  |  | V |
| A0A0G2JLI4 | Probable ATP-dependent RNA helicase DDX5 |  |  | V |
| B7Z1C7 | cDNA FLJ59451, highly similar to Scaffold attachment factor B |  |  | V |
| D3DQF6  POU4F3 | POU domain, class 4, transcription factor 3 |  |  | V |
| A0A384N6G7 | N-acetyl-D-glucosamine kinase |  |  | V |
| K7EMD9 | Keratin, type I cytoskeletal 13 ; KRT13 |  |  | V |
| A0A5C2FYT3 | HUMAN IGL c1326_light_IGKV1-39_IGKJ1 |  |  | V |
| B8QGS9  PKP2 | Truncated plakophilin-2 |  |  | V |
| M0R117 | 60S ribosomal protein L18a |  |  | V |
| E2RVJ0  SLC4A1 | Anion exchange protein |  |  | V |
| A0A7S5BZV4 | IGH c954_heavy_IGHV3-23_IGHD3-10_IGHJ3 |  |  | V |
| A0A2R8Y5T0 | Cytoplasmic dynein 1 heavy chain 1 |  |  | V |
| A0A5C2G2T3 | IGL c3224_light_IGKV3-11_IGKJ4 |  |  | V |
| S6BAM6 | IgG H chain |  |  | V |
| A0A024R580  CAPN1 | Calpain-1 catalytic subunit |  |  | V |
| D1MQ08 | Small conductance calcium activated potassium channel type 4 transcript variant 2; KCNN4 |  |  | V |
| IGL1 | Immunoglobulin lambda-1 light chain |  |  | V |
| A0A0K0K1H9 | Epididymis secretory protein Li 48 |  |  | V |
| P18085 | ADP-ribosylation factor 4 |  |  | V |
| H3BRG4 | Cytochrome b-c1 complex subunit 2, mitochondrial |  |  | V |
| A0A024R2Q4 | Ribosomal protein L15 |  |  | V |
| A0A5C2G4D6 | IGL c4161_light_IGKV4-1_IGKJ2 (Fragment) |  |  | V |
| Q0VG55  TNS1 | TNS1 protein |  |  | V |
| Q71DI3 | Histone H3.2 |  |  | V |
| Q5TEC6 | Histone H3-7 |  |  | V |
| H3-3A  A0A590UJJ6 | Histone H3; H3-3A |  |  | V |
| A0A5C2GQ42 | IG c1003_heavy_IGHV3-23_IGHD3-16_IGHJ4 (Fragment) |  |  | V |
| B7Z4C3 | cDNA FLJ50805, highly similar to Erythrocyte membrane protein band 4.2 |  |  | V |
| B3KNB4 | cDNA FLJ14168 fis, clone NT2RP2001440, highly similar to 14-3-3 protein gamma |  |  | V |
| B2R7Y0 | cDNA, FLJ93654, highly similar to Homo sapiens serpin peptidase inhibitor, clade B (ovalbumin), member 2 (SERPINB2), mRNA |  |  | V |
| A0A384P5S0 | Septin11 |  |  | V |
| D0PNI1 | Epididymis luminal protein 4; YWHAZ |  |  | V |
| H0YLR3 | U2 small nuclear ribonucleoprotein A' (Fragment); SNRPA1 |  |  | V |
| A0A024R571 | EH domain-containing protein 1 |  |  | V |
| Q65ZC9 | Single-chain Fv (Fragment) |  |  | V |
| A0A5C2G2D6 | IGL c2740_light_IGKV1-39_IGKJ5 |  |  | V |
| P05164-2  MPO | Isoform H14 of Myeloperoxidase |  |  | V |
| Q70CQ2  UBP34 | Ubiquitin carboxyl-terminal hydrolase 34 |  |  | V |
| D6RAT0 | 40S ribosomal protein S3a; RPS3A |  |  | V |
| P62906 | 60S ribosomal protein L10a |  |  | V |
| P0C0L4-2  C4A | Isoform 2 of Complement C4-A |  |  | V |
| A0A120HG46 | GCT-A10 heavy chain variable region |  |  | V |
| A0A2R8Y440 | Transcription activator BRG1 |  |  | V |
| A0A024R8S5 | Protein disulfide-isomerase |  |  | V |
| H0Y4R2 | NADPH--hemoprotein reductase |  |  | V |
| Q96HE7 | ERO1-like protein alpha |  |  | V |
| B7Z1D3 | cDNA FLJ54585 |  |  | V |
| A0A5C2GPN1 | IG c1355_heavy_IGHV4-4_IGHD2-2_IGHJ1 |  |  | V |
| A8K3C3 | T-complex protein 1 subunit delta |  |  | V |
| B4DF70 | cDNA FLJ60461, highly similar to Peroxiredoxin-2 |  |  | V |
| B3KM97 | Very-long-chain enoyl-CoA reductase |  |  | V |
| A0A024R4M8 | Retinol dehydrogenase 13 (All-trans and 9-cis) |  |  | V |
| BMP2K | BMP-2-inducible protein kinase |  |  | V |
| A0A5C2GQ13 | IG c1298_heavy_IGHV3-49_IGHD3-3_IGHJ4 |  |  | V |
| A0A5C2G1W4 | IGL c2540_light_IGKV3-20_IGKJ1 |  |  | V |
| A0A2Z4N5U1  AbLIM1 | AbLIM1 |  |  | V |
| G3V5N8 | Zinc finger FYVE domain-containing protein 1; ZFYVE1 |  |  | V |
| F8WB72 | 60S ribosomal protein L35a; RPL35A |  |  | V |
| A0A3B3IRW1 | Tissue factor pathway inhibitor 2 |  |  | V |
| A2RTY6  ITIH2 | Inter-alpha (Globulin) inhibitor H2 |  |  | V |
| A0A5C2GDT0 | IGH c131_heavy__IGHV1-69_IGHD5-24_IGHJ6 |  |  | V |
| Q2NLD4  PURA | PURA protein (Fragment) |  |  | V |
| Q9GZM7-3  TINAGL1 | Isoform 3 of Tubulointerstitial nephritis antigen-like |  |  | V |
| A0A3B3IT57 | NADH dehydrogenase [ubiquinone] 1 beta subcomplex subunit 9 |  |  | V |
| Q5VVD0 | 60S ribosomal protein L11; RPL11 |  |  | V |
| A0A024QYT5 | Serpin peptidase inhibitor, clade E (Nexin, plasminogen activator inhibitor type 1),  member 1; SERPINE1 |  |  | V |
| B3KQC8 | cDNA FLJ90233 fis, clone NT2RM2000514, highly similar to F-box only protein 21 |  |  | V |
| Q4KMQ4  ZFAT | ZFAT protein |  |  | V |
| A0A024R443  DNPEP | Aspartyl aminopeptidase |  |  | V |
| D6RBJ7 | Vitamin D-binding protein |  |  | V |
| P48059 | LIM and senescent cell antigen-like-containing domain protein 1; LIMS1 |  |  | V |
| H0YMV8 | 40S ribosomal protein S27 |  |  | V |
| H0YBE2 | Carbonic anhydrase |  |  | V |
| J3KS25 | RNA helicase; EIF4A1 |  |  | V |
| B3KY12 | cDNA FLJ46581 fis, clone THYMU3043200, highly similar to Splicing factor 3A subunit 3 |  |  | V |
| B2R9T9 | cDNA, FLJ94551 |  |  | V |
| A0A0C4DH52 | Constitutive coactivator of PPAR-gamma-like protein 1; FAM120A |  |  | V |
| B2R5W2 | cDNA, FLJ92657, highly similar to Homo sapiens heterogeneous nuclear ribonucleoprotein C (C1/C2) (HNRPC), transcript variant 2, mRNA |  |  | V |
| A0A024R6P0 | Serpin peptidase inhibitor, clade A (Alpha-1 antiproteinase, antitrypsin), member 3; SERPINA3 |  |  | V |
| A0A2R8Y5B3 | Band 4.1-like protein 2; EPB41L2 |  |  | V |
| A4D0W4 | Aminoadipate-semialdehyde synthase |  |  | V |
| A0A140VK70 | 26S proteasome regulatory subunit 7 |  |  | V |
| A0A024R2F9 | Transmembrane protein 43 isoform 1 |  |  | V |
| A0A2R8Y7R9 | Liprin-alpha-1 |  |  | V |
| I3L3T1 | 14-3-3 protein epsilon |  |  | V |
| P80723-2  BASP1 | Isoform 2 of Brain acid soluble protein 1 |  |  | V |
| A0A3G9HN97 | ATP-dependent RNA helicase DDX60 |  |  | V |
| Q59EY3 | Sp1 transcription factor variant |  |  | V |
| A0A087X0X3 | Heterogeneous nuclear ribonucleoprotein M |  |  | V |
| A0A1L1UHR1 | Voltage-dependent  anion-selective channel protein 1; VDAC1 |  |  | V |
| O15173-2  PGRMC2 | Isoform 2 of Membrane-associated progesterone receptor component 2 |  |  | V |
| Q6IBT3  CCT7 | T-complex protein 1 subunit eta |  |  | V |
| Q2TU64 | T-complex protein 1 subunit gamma |  |  | V |
| H7BYU9 | UPF0687 protein C20orf27 |  |  | V |
| A0A024R3Z5  LANCL1 | LanC lantibiotic synthetase component C-like 1 |  |  | V |
| Q86YS6 | Ras-related protein Rab-43 |  |  | V |
| Q9NR12 | PDZ and LIM domain protein 7; PDLIM7 |  |  | V |
| B4DL14 | ATP synthase subunit gamma |  |  | V |
| E9PP73 | Coatomer subunit beta; COPB1 |  |  | V |
| F2YGG7 | Receptor protein-tyrosine kinase |  |  | V |
| H3BPZ1 | Very-long-chain (3R)-3-hydroxyacyl-CoA dehydratase; HACD3 |  |  | V |
| A0A5C2G8V2 | IGH c364_heavy__IGHV1-3_IGHD3-3_IGHJ5 |  |  | V |
| P61964 | WD repeat-containing protein 5; WDR5 |  |  | V |
| A0A024R7I7 | Ras-related protein Rab-3; RAB3A |  |  | V |
| T2DQ69 | MAP3K3/DDX42 fusion protein 1 |  |  | V |
| Q86XP3 | ATP-dependent RNA helicase DDX42 |  |  | V |
| A7E2A6  TENC1 | Tensin like C1 domain containing phosphatase (Tensin 2); TENC1 |  |  | V |
| Q8WX93-3  PALLD | Isoform 3 of Palladin |  |  | V |
| A0A024R5Z8 | RAB11A, member RAS oncogene family |  |  | V |
| A0A024R9L6 | ST3 beta-galactoside alpha-2,3-sialyltransferase 1; ST3GAL1 |  |  | V |
| A0A2X0SFJ0  TRIO | Non-specific  serine/threonine protein kinase |  |  | V |
| Q8NFW8-2  CMAS | Isoform 2 of N-acylneuraminate  cytidylyltransferase |  |  | V |
| A0A7P0T9F7  SLC3A2 | 4F2 cell-surface antigen heavy chain |  |  | V |
| A0A024R842  PAPPA | Pregnancy-associated  plasma protein A, pappalysin 1 |  |  | V |
| A0A3B3IRZ4 | IQ motif and SEC7 domain-containing protein 1; IQSEC1 |  |  | V |
| A0A024R4Z1 | HCG2042749; E3 ubiquitin-protein ligase ZFP91 |  |  | V |
| Q14213 | Interleukin-27 subunit beta; EBI3 |  |  | V |
| A8K7B1 | cDNA FLJ78384, highly similar to Homo sapiens RUN and FYVE domain containing 1 (RUFY1), mRNA |  |  | V |
| B0QZK4 | eterochromatin protein 1-binding protein 3; HP1BP3 |  |  | V |
| P07305-2  H1-0 | Isoform 2 of Histone H1.0 |  |  | V |
| B3KQV6 | Serine/threonine-protein phosphatase 2A 65 kDa regulatory subunit A alpha isoform; PPP2R1A |  |  | V |
| A0A7S5C185 | IGH c1972_heavy_IGHV4-39_IGHD6-25_IGHJ4 |  |  | V |
| Q2TNI1 | Caveolin |  |  | V |
| A0A024R374 | Cathepsin B |  |  | V |
| Q4ZG32  EPB41L5 | Band 4.1-like protein 5 |  |  | V |
| G3V4U2 | Tubulin beta-3 chain; TUBB3 |  |  | V |
| D3DNS0  ACTL6A | Actin-like 6A |  |  | V |
| A0A7P0S5H5  RPS20 | 40S ribosomal protein S20 |  |  | V |
| Q9UMS4 | Pre-mRNA-processing factor 19 |  |  | V |
| L7RSL3 | Receptor protein-tyrosine kinase |  |  | V |
| Q7Z6U0 | TATA box-binding protein-like 1 |  |  | V |
| Q8NDL6 | Uncharacterized protein DKFZp434I2435 |  |  | V |
| O00425 | Insulin-like growth factor 2 mRNA-binding protein 3; IGF2BP3 |  |  | V |
| A0A024R4E5 | High density lipoprotein binding protein (Vigilin) |  |  | V |
| H0Y449 | Y-box-binding protein 1 |  |  | V |
| Q5TF85 | Polynucleotide Adenylyltransferase; TENT5A |  |  | V |
| A0A024QZY5 | Serine/threonine-protein kinase PRP4 homolog |  |  | V |
| A0A5C2GSW6 | IG c1580_heavy_IGHV1-69_IGHD6-6_IGHJ4 |  |  | V |
| A0A024R2L1 | WD repeat-containing protein 48 |  |  | V |
| V9HW21 | Carbonic anhydrase; HEL-76 |  |  | V |
| Q53R19 | Arp2/3 complex 34 kDa subunit; ARPC2 |  |  | V |
| O43290 | U4/U6.U5 tri-snRNP-associated protein 1 |  |  | V |
| A0A7S5BYK7 | IGH c838_heavy_IGHV3-7_IGHD4-17_IGHJ5 |  |  | V |
| A0A024QZV0 | Protein disulfide-isomerase |  |  | V |
| Q9UDW1 | Cytochrome b-c1 complex subunit 9 |  |  | V |
| A0A024R3W5 | Solute carrier family 39 (Zinc transporter), member 10 |  |  | V |
| A0A384MTQ3 | Adenosylhomocysteinase |  |  | V |
| A0A0A0MTH3 | Integrin-linked protein kinase |  |  | V |
| A0A384MTW7 | Nucleoside diphosphate kinase |  |  | V |
| A0A5C2GAC5 | IGH + IGL c72_heavy_IGHV3-23_IGHD5-24_IGHJ5 |  |  | V |
| P10696 | Alkaline phosphatase, germ cell type; ALPG |  |  | V |
| Q1W6G9  HBZ | Hemoglobin subunit zeta |  |  | V |
| A0A024R1Q8 | 60S ribosomal protein L23; RPL23 |  |  | V |
| B3KQG6 | cDNA FLJ90427 fis, clone NT2RP3000481, highly similar to Importin-7 |  |  | V |
| B4DN77  CAPN2 | Calpain-2 catalytic subunit |  |  | V |
| A0A2R8YFH5 | Protein transport protein SEC23 |  |  | V |
| A0A024R2G7 | Ribosomal protein L32 |  |  | V |
| O94919 | Endonuclease domain-containing 1 protein |  |  | V |
| A0A0A0MRF3 | Transcriptional enhancer factor TEF-3 |  |  | V |
| A0A384MEG1 | Fascin |  |  | V |
| ST2B1 | Isoform 2 of Sulfotransferase 2B1 |  |  | V |
| B5MD38 | Trifunctional enzyme subunit beta, mitochondrial |  |  | V |
| A0A024QZK8 | Heterogeneous nuclear ribonucleoprotein H3 (2H9) |  |  | V |
| A0A3B3ITC6 | Segment polarity protein dishevelled homolog DVL-3 |  |  | V |
| P28074 | Proteasome subunit beta type-5 |  |  | V |
| M0QXL5 | rRNA 2'-O-methyltransferase fibrillarin |  |  |  |
| A0A024RB22 | SWI/SNF related, matrix associated, actin dependent regulator of chromatin, Subfamily c, member 2 |  |  | V |
| B1Q2B0  URCC5 | URCC5 |  |  | V |
| A0A140VJW5 | Aspartate—Trna ligase, cytoplasmic |  |  | V |
| A0A0C4DGG1 | Protein kinase C and casein kinase substrate in neurons protein 3; PACSIN3 |  |  | V |
| A0A024R9W7 | Transmembrane protein 33; TMEM33 |  |  | V |
| A0A024R3Y6  IDH1 | Isocitrate dehydrogenase [NADP] |  |  | V |
| Q8NBH6  FBLN1 | Fibulin-1 |  |  | V |
| A0A1B0GVS3 | Alpha-centractin |  |  | V |
| A0A0S2Z4V6 | Wolfram syndrome 1 isoform 1 |  |  | V |
| A0A0B4J2B4 | 40S ribosomal protein S15; RPS15 |  |  | V |
| B2R4W8 | 40S ribosomal protein S15a |  |  | V |
| Q9BU76-4  MMTAG2 | Isoform 4 of Multiple myeloma tumor-associated protein 2 |  |  | V |
| Q53HB3 | Proteasome 26S ATPase subunit 1 variant |  |  | V |
| P62318-2  SNRPD3 | Isoform 2 of Small nuclear ribonucleoprotein Sm D3; SNRPD3 |  |  | V |
| A0A024RAB9 | Adiponectin A |  |  | V |
| A4UCS6  PRDX6 | Peroxiredoxin 6 |  |  | V |
| A0A024R5C2 | 2'-phosphotransferase; TRPT1 |  |  | V |
| P61160 | Actin-related protein 2 |  |  | V |
| P04899-4  GNAI2 | Isoform sGi2 of Guanine nucleotide-binding protein G(i) subunit alpha-2 |  |  | V |
| B2RBR9  KPNB1 | cDNA, FLJ95650, highly similar to Homo sapiens karyopherin (importin) beta 1 (KPNB1) |  |  | V |
| V9HW88 | Calreticulin |  |  | V |
| B4DRS6 | Sidoreflexin |  |  | V |
| A0A024R1A3 | E1 ubiquitin-activating enzyme |  |  | V |
| A0A024R223  VPS13A | Vacuolar protein sorting 13A |  |  | V |
| A0A024RDE8  PDLIM5 | PDZ and LIM domain 5 |  |  | V |
| A0A0S2Z3Y1 | Galectin-3-binding protein; LGALS3BP |  |  | V |
| A0A024R5P0 | Non-specific serine/threonine protein kinase; PAK1 |  |  | V |
| A0A343H8S5 | Cytochrome c oxidase subunit 2; COX2 |  |  | V |
| A0A024RAR8  ARTS-1 | Aminopeptidase; ARTS-1 |  |  | V |
| H0Y5P0 | Forkhead-associated domain-containing protein 1 |  |  | V |
| P0DML2 | Chorionic somatomammotropin hormone 1 |  |  | V |
| E5KLJ9 | Dynamin-like 120 kDa protein, mitochondrial |  |  | V |
| A0A140VK56 | Transaldolase |  |  | V |
| A0A087WU68 | Polypyrimidine tract-binding protein 1 |  |  | V |
| A0A2R8YF49  ARHGEF5 | Rho guanine nucleotide exchange factor 5 |  |  | V |
| Q6ZMF1  GLG1 | Golgi apparatus protein 1 |  |  | V |
| A0A087X2D0 | Serine/arginine-rich-splicing factor 3 |  |  | V |
| A0A024R0J9 | Heterogeneous nuclear ribonucleoprotein U-like 1; HNRPUL1 |  |  | V |
| G3V2E7 | Kinesin light chain |  |  | V |
| A0A024R1U4 | RAB5C, member RAS oncogene family |  |  | V |
| F5GWF6 | T-complex protein 1 subunit beta |  |  | V |
| A0A024RDL1  CCT6A | Chaperonin containing TCP1, subunit 6A (Zeta 1) |  |  | V |
| B5BTZ8  SNRPB2 | Small nuclear ribonucleoprotein polypeptide B'' |  |  | V |
| A0A158RFU6 | RAB7, member RAS oncogene family |  |  | V |
| A0A2X0TVY0 | FARP1 (Fragment) |  |  | V |
| Q15075 | Early endosome antigen 1 |  |  | V |
| J3KMX2 | SWI/SNF-related matrix-associated actin-dependent regulator of chromatin subfamily D member 2; SMARCD2 |  |  | V |
| A0A024R6S1 | DnaJ (Hsp40) homolog, subfamily A, member 2; DNAJA2 |  |  | V |
| Q07954 | Pro-low-density lipoprotein receptor-related protein 1; LRP1 |  |  | V |
| A0A024R326  RPL29 | 60S ribosomal protein L29; RPL29 |  |  | V |
| A0A024R3J7 | Dolichyl-diphosphooligosaccharide--protein glycotransferase |  |  | V |
| A0A7I2V2S8  NCL | Nucleolin; NCL |  |  | V |
| A0A024RDV7 | Importin subunit alpha; KPNA3 |  |  | V |
| D6RIA3 | Uncharacterized protein C4orf54 |  |  | V |
| A0A1B0GUF4 | Ecotropic viral  integration site 5 protein homolog |  |  | V |
| B2R4C1  RPL31 | 60S ribosomal protein L31 |  |  | V |
| P02760 | Protein AMBP |  |  | V |
| H0YAR3 | DNA topoisomerase I, Mitochondrial; TOP1MT |  |  | V |
| A0A140VK43 | Proteasome subunit alpha type |  |  | V |
| M0R210 | 40S ribosomal protein S16 |  |  | V |
| A0A8I5KUI4  LARP7 | La-related protein 7; LARP7 |  |  | V |
| A0A140VKE9  DYNC1I2 | Testis tissue sperm-binding protein Li 66n |  |  | V |
| A0A024QZA9 | Protein-serine/threonine kinase; BCKDK |  |  | V |
| Q9Y6L7 | Tolloid-like protein 2; TLL2 |  |  | V |
| H7C571 | Transcription cofactor vestigial-like protein 3 |  |  | V |
| A0A024R8V0 | Septin 9; SEPT9 |  |  | V |
| H0YAS9 | ER membrane protein complex subunit 2; EMC2 |  |  | V |
| A0A024R5M3 | Cortactin; CTTN |  |  | V |
| A0A3B3ITX4 | Uncharacterized protein |  |  | V |
| F5H3Y4 | Probable ATP-dependent RNA helicase DHX37 |  |  | V |
| A0A087WWI6 | DDB1- and CUL4-associated factor 7 |  |  | V |
| B3KUB9  SWAP70 | SWAP-70 protein |  |  | V |
| A0A087WU53 | Magnesium transporter protein 1; MAGT1 |  |  | V |
| B5BUB5  SSB | Autoantigen La; SSB |  |  | V |
| B4DN40 | cDNA FLJ54368, highly similar to Phosphoglucomutase-2 |  |  | V |
| D6W648 | HCG2004001 |  |  | V |
| A0A5F9ZH11  BRPF1 | Peregrin |  |  | V |
| A0A2R8Y4L2 | Heterogeneous nuclear ribonucleoprotein A1-like 3; HNRNPA1L3 |  |  | V |
| P59044-2  NLRP6 | Isoform 2 of NACHT, LRR and PYD domains-containing protein 6 |  |  | V |
| Q7Z5T5 | EIF3A ; eIF3A protein |  |  | V |
| A0A0J9YW64 | Dedicator of cytokinesis protein 11; DOCK11 |  |  | V |
| Q14520-2  HABP2 | Isoform 2 of Hyaluronan-binding protein 2 |  |  | V |
| A0A024R4K3  MDH2 | Malate dehydrogenase |  |  | V |
| A0A096LP62 | Inter-alpha-trypsin inhibitor heavy chain H5 |  |  | V |
| H9ZYJ2 | Thioredoxin |  |  | V |
| Q5NDL2-2  EOGT | Isoform 2 of EGF domain-specific O-linked N-acetylglucosamine transferase |  |  | V |
| A0A024RB75 | Citrate synthase, mitochondrial |  |  | V |
| A0A804HK18  SACS | Sacsin; SACS |  |  | V |
| A0A140VK45  PSMB1 | Proteasome subunit beta |  |  | V |
| A0A7I2V2H0  XPO1 | Exportin-1 |  |  | V |
| E9PK85 | Ras-related protein R-Ras2; RRAS2 |  |  | V |
| H7BZK6 | Ubiquitin carboxyl-terminal hydrolase 46; USP46 |  |  | V |
| Q8N4E4 | Phosducin-like protein 2; PDCL2 |  |  | V |
| A0A024R8K7  ITGB4 | Integrin beta; ITGB4 |  |  | V |
| B4DP62 | Solute carrier family 25 (Mitochondrial carrier citrate transporter), member 1; SLC25A1 |  |  | V |
| A0A024R7F1  PRKCSH | Glucosidase 2 subunit beta |  |  | V |
| B4DWH6  SLC39A6 | cDNA FLJ50261, highly similar to Zinc transporter SLC39A6 |  |  | V |
| A0A8I5KRA4  MYO5A | Unconventional myosin-Va |  |  | V |
| A4FU77  SNRNP200 | SNRNP200 protein |  |  | V |
| A0A024R7V6 | RAB2, member RAS oncogene family |  |  | V |
| A4QMX9  C2orf16 | C2orf16 protein |  |  | V |
| H0YDT6 | Eukaryotic translation initiation factor 3 subunit F; EIF3F |  |  | V |
| A8K9F2  ZNF652 | cDNA FLJ75819, highly similar to Homo sapiens zinc finger protein 652 (ZNF652), mRNA |  |  | V |
| A0A140VJJ8  GNB1 | Guanine nucleotide binding protein (G protein), beta polypeptide 1 |  |  | V |
| A0A2R8YCS7 | Deoxynucleoside triphosphate triphosphohydrolase SAMHD1 |  |  | V |
| A0A024RC06 | Myotubularin; MTM1 |  |  | V |
| A0A140VK35 | 1-phosphatidylinositol 4-kinase |  |  | V |
| A0A8I5KQF7  NDUFB11 | NADH dehydrogenase  [ubiquinone] 1 beta subcomplex subunit 11, mitochondrial; NDUFB11 |  |  |  |
| A0A384NKJ3 | Epididymis secretory sperm binding protein |  |  | V |
| A0A590UJS4  NIPBL | Nipped-B protein |  |  | V |
| D3DU92 | RNA-binding protein with serine-rich domain 1; RNPS1 |  |  | V |
| A0A087WTQ3 | Cytoplasmic FMR1-interacting protein 2; CYFIP2 |  |  | V |
| B3KX47  LLGL2 | cDNA FLJ44733 fis, clone  BRACE3026290, highly similar to Lethal(2) giant larvae protein homolog |  |  | V |
| K7EJ44 | Profilin; PFN1 |  |  | V |
| Q6ICQ8 | ARHG protein |  |  | V |
| Q5HYJ3-2  FAM76B | Isoform 2 of Protein FAM76B |  |  | V |
| A0A0S2Z499  RBMS1 | RNA binding motif single stranded interacting protein 1 isoform 1; RBMS1 |  |  | V |
| B4DRG0  HDAC1 | DNA FLJ51764, highly similar  to Histone deacetylase 1 |  |  | V |
| L0R5A1  CSF2RB | Alternative protein CSF2RB |  |  | V |
| A0A087WUL2 | Proteasome subunit beta type-3; PSMB3 |  |  | V |
| A0A140VJP5 | S-adenosylmethionine synthase |  |  | V |
| A0A1W2PPH1 | Malic enzyme; ME2 |  |  | V |
| A0A7I2V2M2  ACBD5 | Acyl-CoA-binding domain-containing protein 5 |  |  | V |
| D3DUU0  CD163 | CD163 antigen |  |  | V |
| A0A7I2V3U3  EIF3I | Eukaryotic translation initiation factor 3 subunit I |  |  | V |
| B4DJI2  GRN | cDNA FLJ53342, highly similar to Granulins |  |  | V |
| B4DLR4  HSPA12B | cDNA FLJ55280, highly similar  to Heat shock 70 kDa protein 12B |  |  | V |
|  |  |  |  |  |
