# Supplementary material for: Placental Protein Citrullination Signatures Are Modified in Early- and Late-Onset Fetal Growth Restriction
Source: Int J Mol Sci. 2025 Apr 29;26(9):4247. doi: 10.3390/ijms26094247 (PMC12071715; doi:10.3390/ijms26094247)
Supplement: Supplementary file 1 [file ijms-26-04247-s001.zip › Supplementary Table S2.docx]

**Supplementary Table S2.** KEGG and DISEASES pathways associated with the placental citrullinomes. A tick (V) indicates that the pathway was present in the placental citrullinome of the group. Control (AGA), early-onset FGR (E-FGR) and late-onset FGR (L-FGR) samples.

| **KEGG DESCRIPTION** | **Control AGA** | **E-FGR** | **L-FGR** |
| --- | --- | --- | --- |
| Steroid hormone biosynthesis | V |  |  |
| Ovarian steroidogenesis | V |  |  |
| Oestrogen signalling pathway | V |  |  |
| Legionellosis | V | V | V |
| Ribosome | V | V | V |
| Spliceosome | V | V | V |
| Protein processing in endoplasmic reticulum | V | V | V |
| Complement and coagulation cascades | V | V | V |
| Prion disease | V | V | V |
| Amyotrophic lateral sclerosis | V | V | V |
| Antigen processing and presentation | V | V |  |
| Systemic lupus erythematosus | V |  | V |
| Bacterial invasion of epithelial cells |  | V | V |
| Amoebiasis |  | V | V |
| Tight junction |  | V | V |
| Proteasome |  | V | V |
| Proteoglycans in cancer |  | V | V |
| Focal adhesion |  | V | V |
| Parkinson’s disease |  | V | V |
| Spinocerebellar ataxia |  | V | V |
| Regulation of actin skeleton |  | V | V |
| Phagosome |  | V | V |
| Viral carcinogenesis |  | V | V |
| Huntington’s disease |  | V | V |
| Endocytosis |  | V | V |
| Alzheimer’s disease |  | V | V |
| Adherens junction |  | V | V |
| Leucocyte transendothelial migration |  | V |  |
| Thyroid hormone synthesis |  | V |  |
| Hypertrophic cardiomyopathy |  | V |  |
| ECM receptor interaction |  | V |  |
| Arrhythmogenic right ventricular cardiomyopathy |  | V |  |
| Disease by infectious agent |  | V |  |
| Citrate cycle (TCA cycle) |  |  | V |
| *S.aureus* infection |  |  | V |
| Antigen processing and presentation |  |  | V |
| Leukocyte transendothelial migration |  |  | V |
| Salmonella infection |  |  | V |
| Carbon metabolism |  |  | V |
| Shigellosis |  |  | V |
| Hippo signalling pathway |  |  | V |
| Yersinia infection |  |  | V |
| **DISEASES** | **Control** | **E-FGR** | **L-FGR** |
| Primary cutaneous amyloidosis | V | V |  |
| Amyloidosis | V | V | V |
| Familial visceral amyloidosis |  | V | V |
| Diamond-Blackfan anaemia |  | V | V |
| Anaemia |  | V | V |
| Hematopoietic system disease |  | V | V |
| Autosomal dominant disease |  | V | V |
| Disease |  | V | V |
| Congenital hypoplastic anaemia |  |  | V |
| Autosomal dominant non-syndromic intellectual disability |  |  | V |
| Haemolytic anaemia |  |  | V |
| Cardiovascular system disease |  |  | V |
| Physical disorder |  |  | V |
| Inherited metabolic disorder |  |  | V |
| Genetic disease |  |  | V |
| Disease of anatomical entity |  |  | V |
