# Supplementary material for: Placental Protein Citrullination Signatures Are Modified in Early- and Late-Onset Fetal Growth Restriction
Source: Int J Mol Sci. 2025 Apr 29;26(9):4247. doi: 10.3390/ijms26094247 (PMC12071715; doi:10.3390/ijms26094247)
Supplement: Supplementary file 1 [file ijms-26-04247-s001.zip › Supplementary Table S3.docx]

**Supplementary Table S3:** Human Phenotypes (Monarch) associated with the placental citrullinomes. A tick (V) indicates that the pathway was present in the placental citrullinome of the group. Control (AGA), early-onset FGR (E-FGR) and late-onset FGR (L-FGR) samples.

| **Human Phenotype (Monarch)** | **Control** | **E-FGR** | **L-FGR** |
| --- | --- | --- | --- |
| Anaemia of inadequate production | V | V | V |
| Pain | V | V | V |
| Abnormal cardiovascular system physiology |  | V | V |
| Abnormality of the skin |  | V | V |
| Abnormality of the integument |  | V | V |
| Congestive heart failure |  | V | V |
| Abnormality of the respiratory system |  | V | V |
| Abnormality of skin morphology |  | V | V |
| Abnormality of blood and blood-forming tissues |  | V | V |
| Pulmonary arterial hypertension |  | V | V |
| Oedema |  | V | V |
| Abnormality of fluid regulation |  | V | V |
| Abnormality of immune system physiology |  | V | V |
| Abnormal skeletal morphology |  | V | V |
| Abnormal homeostasis |  | V | V |
| Abnormality of the skeletal system |  | V | V |
| Phenotypic abnormality |  | V | V |
| Abnormality of the cardiovascular system |  | V | V |
| Abnormality of metabolism/homeostasis |  | V | V |
| Abnormality of the immune system |  | V | V |
| Abnormality of the digestive system |  | V | V |
| Small for gestational age |  | V | V |
| Abnormal muscle physiology |  | V | V |
| Abnormal respiratory system physiology |  | V | V |
| Abnormality of the musculoskeletal system |  | V | V |
| Aplasia/Hypoplasia of the cerebrum |  | V | V |
| Abnormality of the musculature |  | V | V |
| Microcephaly |  | V | V |
| Decreased head circumference |  | V | V |
| Abnormal heart valve physiology |  | V | V |
| Abnormality of the urinary system |  | V | V |
| Abnormality of head or neck |  | V | V |
| Abnormality of prenatal development or birth |  | V | V |
| Unusual infection |  | V | V |
| Abnormal oral cavity morphology |  | V | V |
| Abnormality of the kidney |  | V | V |
| Abnormality of cardiovascular system morphology |  | V | V |
| Abnormality of skull size |  | V | V |
| Abnormal heart morphology |  | V | V |
| Abnormality of the head |  | V | V |
| Abnormality of the forehead |  | V | V |
| Abnormality of the eye |  | V | V |
| Abnormal vascular physiology |  | V | V |
| Sprengel anomaly |  | V | V |
| Abnormal facial shape |  | V | V |
| Decreased body weight |  | V | V |
| Abnormality of the upper urinary tract |  | V | V |
| Abnormal eye morphology |  | V | V |
| Abnormal nervous system morphology |  | V | V |
| Abnormality of the mouth |  | V | V |
| Cholestasis |  | V | V |
| Dyspnea |  | V | V |
| Abnormal central motor function |  | V | V |
| Abnormality of the nervous system |  | V | V |
| Dilated cardiomyopathy |  | V | V |
| Abnormality of skin adnexa morphology |  | V | V |
| Dermatological manifestations of systemic disorders |  | V | V |
| Recurrent infections |  | V | V |
| Abnormal axial skeleton morphology |  | V | V |
| Abnormal platelet count |  | V | V |
| Abnormal atrioventricular valve physiology |  | V | V |
| Anaemia |  | V | V |
| Growth delay |  | V | V |
| Abnormal eye physiology |  | V | V |
| Synophrys |  | V | V |
| Abnormal circulating metabolite concentration |  | V | V |
| Abnormality of the vasculature |  | V | V |
| Abdominal symptom |  | V | V |
| Abnormal skeletal muscle morphology |  | V | V |
| Abnormal nervous system physiology |  | V | V |
| Abnormal skull morphology |  | V | V |
| Abnormal muscle tone |  | V | V |
| Increased inflammatory response |  | V | V |
| Abnormal systemic blood pressure |  | V | V |
| Thin vermilion border |  | V | V |
| Strabismus |  | V | V |
| Thrombocytopenia |  | V | V |
| Abnormality of neutrophils |  | V | V |
| Abnormality of the lower limb |  | V | V |
| Muscle fiber atrophy |  | V | V |
| Abnormal skin morphology of the palm |  | V | V |
| Abnormality of the face |  | V | V |
| Abnormal hair morphology |  | V | V |
| Facial hypertrichosis |  | V | V |
| Abnormal lip morphology |  | V | V |
| Morphological central nervous system abnormality |  | V | V |
| Abnormal renal morphology |  | V | V |
| Cardiomyopathy |  | V | V |
| Abnormal upper lip morphology |  | V | V |
| Aplasia/Hypoplasia involving the central nervous system |  | V | V |
| Abnormal palm morphology |  | V | V |
| Pallor |  | V | V |
| Atrioventricular valve regurgitation |  | V | V |
| Abnormality of the orbital region |  | V | V |
| Sign or symptom |  | V | V |
| Abnormality of the philtrum |  | V | V |
| Abnormal pinna morphology |  | V | V |
| Abnormality of the lower urinary tract |  | V | V |
| Hypospadias |  | V | V |
| Respiratory insufficiency |  | V | V |
| Abnormality of brain morphology |  | V | V |
| Abnormal circulating protein concentration |  | V | V |
| Global developmental delay |  | V | V |
| Generalized abnormality of skin |  | V | V |
| Abnormal joint morphology |  | V | V |
| Generalized amyloid deposition |  | V | V |
| Feeding difficulties |  | V | V |
| Constitutional symptom |  | V | V |
| Abnormal pattern of respiration |  | V | V |
| Haematuria |  | V | V |
| Foot joint contracture |  | V | V |
| Regional abnormality of skin |  | V | V |
| Abnormality of digestive system physiology |  | V | V |
| Abnormal ocular adnexa morphology |  | V | V |
| Abnormal cardiac septum morphology |  | V | V |
| Abnormal thrombosis |  | V | V |
| Abnormal renal physiology |  | V | V |
| Abnormal bleeding |  | V | V |
| Abnormal eyelid morphology |  | V | V |
| Abnormality of the ear |  | V | V |
| Normochromic anaemia |  | V | V |
| EMG abnormality |  | V | V |
| Paralysis |  | V | V |
| Proximal placement of thumb |  | V | V |
| Absent muscle fiber emerin |  | V | V |
| Hypotonia |  | V | V |
| Abnormality of the biliary system |  | V | V |
| Neurodevelopmental delay |  | V | V |
| Abnormality of the urinary system physiology |  | V | V |
| Abnormal tricuspid valve physiology |  | V | V |
| Behavioural abnormality |  | V | V |
| Myopathy |  | V | V |
| Growth abnormality |  | V | V |
| Abnormality of the genitourinary system |  | V | V |
| Failure to thrive |  | V | V |
| Atrial septal defect |  | V | V |
| Deviation of the thumb |  | V | V |
| Localized skin lesion |  | V | V |
| Abnormal eyebrow morphology |  | V | V |
| Abnormal number of erythroid precursors |  | V | V |
| Abnormality of eye movement |  | V | V |
| Abnormal nephron morphology |  | V | V |
| Abnormality of higher mental function |  | V | V |
| Autism |  | V | V |
| Abnormality of limbs |  | V | V |
| Thrombocytosis |  | V | V |
| Gastrointestinal carcinoma |  | V | V |
| Abnormal cerebral morphology |  | V | V |
| Fetal anomaly |  | V | V |
| High palate |  | V | V |
| Abnormal renal glomerulus morphology |  | V | V |
| Neurodevelopmental abnormality |  | V | V |
| Premature birth |  | V |  |
| Abnormal adipose tissue morphology |  | V |  |
| Lipodystrophy |  | V |  |
| Type 1 muscle fiber atrophy |  | V |  |
| Mitral regurgitation |  | V |  |
| Long palpebral fissure |  | V |  |
| Lipoatrophy |  | V |  |
| Hypotension |  | V |  |
| Decreased calvarial ossification |  | V |  |
| Dysplastic corpus callosum |  | V |  |
| Back pain |  | V |  |
| Decreased cervical spine flexion due to contractures of posterior cervical muscles |  | V |  |
| Obsolete Toe walking |  | V |  |
| Abnormality of cranial sutures |  | V |  |
| Long philtrum |  |  | V |
| Macrocytic anaemia |  |  | V |
| Neoplasm |  |  | V |
| Neoplasm by anatomical site |  |  | V |
| Abnormal vascular morphology |  |  | V |
| Increased mean corpuscular volume |  |  | V |
| Ptosis |  |  | V |
| Abnormal conjugate eye movement |  |  | V |
| Myelodysplasia |  |  | V |
| Erythroid hypoplasia |  |  | V |
| Neoplasm of the genitourinary tract |  |  | V |
| Abnormality of the gastrointestinal tract |  |  | V |
| Persistence of haemoglobin F |  |  | V |
| Abnormality of the ocular adnexa |  |  | V |
| Abnormal erythrocyte morphology |  |  | V |
| Abnormality of the urethra |  |  | V |
| Radial artery aplasia |  |  | V |
| Elevated red cell adenosine deaminase level |  |  | V |
| Abnormal erythrocyte enzyme level |  |  | V |
| Abnormality of body weight |  |  | V |
| Macrocytic dyserythropoietic anaemia |  |  | V |
| Osteosarcoma |  |  | V |
| Neoplasm of the large intestine |  |  | V |
| Pure red cell aplasia |  |  | V |
| Venous thrombosis |  |  | V |
| Neoplasm of the colon |  |  | V |
| Webbed neck |  |  | V |
| Adenocarcinoma of the intestines |  |  | V |
| Abnormal reticulocyte morphology |  |  | V |
| Congenital abnormal hair pattern |  |  | V |
| Abnormal hair pattern |  |  | V |
| Malignant genitourinary tract tumour |  |  | V |
| Aplasia of the fingers |  |  | V |
| Aplasia/Hypoplasia of fingers |  |  | V |
| Abnormality of finger |  |  | V |
| Deviation of finger |  |  | V |
| Abnormal ear morphology |  |  | V |
| Neurological speech impairment |  |  | V |
| Abnormality of the vertebral column |  |  | V |
| Abnormality of body height |  |  | V |
| Abnormality of the hairline |  |  | V |
| Abnormality of the frontal hairline |  |  | V |
| Low anterior hairline |  |  | V |
| Short stature |  |  | V |
| Abnormality of the thenar eminence |  |  | V |
| Short thumb |  |  | V |
| Aplasia/Hypoplasia of the thumb |  |  | V |
| Haematological neoplasm |  |  | V |
| Abnormal haemoglobin |  |  | V |
| Reticulocytopenia |  |  | V |
| Adenocarcinoma of the colon |  |  | V |
| Neoplasm by histology |  |  | V |
| Abnormal enzyme/coenzyme activity |  |  | V |
| Feeding difficulties in infancy |  |  | V |
| Neoplasm of the gastrointestinal tract |  |  | V |
| Developmental glaucoma |  |  | V |
| Abnormal helix morphology |  |  | V |
| Abnormal scapula morphology |  |  | V |
| Abnormality of the upper limb |  |  | V |
| Abnormality of pulmonary circulation |  |  | V |
| Abnormality of blood circulation |  |  | V |
| Acute myeloid leukaemia |  |  | V |
| Partial duplication of thumb phalanx |  |  | V |
| Duplication of thumb phalanx |  |  | V |
| Abnormality of thumb phalanx |  |  | V |
| Absent thumb |  |  | V |
| Abnormality of coordination |  |  | V |
| Abnormal musculoskeletal physiology |  |  | V |
| Abnormal blood vessel morphology |  |  | V |
| Abnormal cerebral vascular morphology |  |  | V |
| Haematological measurement |  |  | V |
| Gait disturbance |  |  | V |
| Gait ataxia |  |  | V |
| Leukaemia |  |  | V |
| Abnormal large intestine morphology |  |  | V |
| Partial duplication of the phalanx of hand |  |  | V |
| Lethargy |  |  | V |
| Abnormality of the musculature of the limbs |  |  | V |
| Abnormality of the scalp hair |  |  | V |
| Reticulocyte measurement |  |  | V |
| Abnormal myocardium morphology |  |  | V |
| Abnormal hair quantity |  |  | V |
| Internal haemorrhage |  |  | V |
| Microtia |  |  | V |
| Neoplasm of the skeletal system |  |  | V |
| Poor suck |  |  | V |
| Acute leukaemia |  |  | V |
| Abnormal immune system morphology |  |  | V |
| Hydrops fetalis |  |  | V |
| Nystagmus |  |  | V |
| Scoliosis |  |  | V |
| Abnormal myelination |  |  | V |
| Vascular skin abnormality |  |  | V |
| Short digit |  |  | V |
| Short finger |  |  | V |
| Abnormal involuntary eye movements |  |  | V |
| Horseshoe kidney |  |  | V |
| Autistic behaviour |  |  | V |
| Ocular anterior segment dysgenesis |  |  | V |
| Abnormality of the neck |  |  | V |
| Abnormality of the curvature of the vertebral column |  |  | V |
| Synophrys |  |  | V |
| Abnormal foot morphology |  |  | V |
| Abnormality of the hand |  |  | V |
| Nonimmune hydrops fetalis |  |  | V |
| Leukopenia |  |  | V |
| Aplasia/hypoplasia of the extremities |  |  | V |
| Abnormal leukocyte morphology |  |  | V |
| Abnormal cellular immune system morphology |  |  | V |
| Complete blood cell count |  |  | V |
| Abnormal penis morphology |  |  | V |
| Abnormality of female external genitalia |  |  | V |
| Cognitive impairment |  |  | V |
| Abnormal fetal morphology |  |  | V |
| Abnormal localization of kidney |  |  | V |
| Abnormal cardiac ventricle morphology |  |  | V |
| Low-set ears |  |  | V |
| Abnormality of the musculature of the hand |  |  | V |
| Abnormality of skin physiology |  |  | V |
| Abnormal cerebral cortex morphology |  |  | V |
| Inflammatory abnormality of the skin |  |  | V |
| Intracranial haemorrhage |  |  | V |
| Functional abnormality of the gastrointestinal tract |  |  | V |
| Abnormal neck morphology |  |  | V |
| Triphalangeal thumb |  |  | V |
| Abnormal calvaria morphology |  |  | V |
| Abnormality of limb bone morphology |  |  | V |
| Abnormality of joint mobility |  |  | V |
| Abnormality of movement |  |  | V |
| Abnormal leukocyte count |  |  | V |
| Leukocyte count |  |  | V |
| Abnormality of the abdominal organs |  |  | V |
| Abnormal thorax morphology |  |  | V |
| Abnormality of the nail |  |  | V |
| Ataxia |  |  | V |
| Clinodactyly |  |  | V |
| Delayed ability to walk |  |  | V |
| Neutropenia |  |  | V |
| Intellectual disability |  |  | V |
| Motor delay |  |  | V |
| Abnormal morphology of the great vessels |  |  | V |
| Pectus excavatum |  |  | V |
| Abnormal location of ears |  |  | V |
| Abnormality of globe location |  |  | V |
| Elbow flexion contracture |  |  | V |
| Elbow contracture |  |  | V |
| Abnormality of the liver |  |  | V |
| Abnormal epidermal morphology |  |  | V |
| Developmental cataract |  |  | V |
| Hypertrichosis |  |  | V |
| Coarctation of aorta |  |  | V |
| Aplasia/hypoplasia involving bones of the hand |  |  | V |
| Abnormal granulocyte morphology |  |  | V |
| Abnormal cerebral ventricle morphology |  |  | V |
| Abnormal aggressive, impulsive or violent behaviour |  |  | V |
| Abnormal appendicular skeleton morphology |  |  | V |
| Abnormality of the voice |  |  | V |
| Abnormality of the dentition |  |  | V |
| Sarcoma |  |  | V |
| Haemangioma |  |  | V |
| Aplasia cutis congenita |  |  | V |
| Aplasia/hypoplasia involving bones of the upper limbs |  |  | V |
| Hypoplasia of the corpus callosum |  |  | V |
| Renal agenesis |  |  | V |
| Abnormal cerebral white matter morphology |  |  | V |
| Abnormal oesophagus physiology |  |  | V |
| Fragile nails |  |  | V |
| Muscle weakness |  |  | V |
| Urinary tract neoplasm |  |  | V |
| Gastrointestinal haemorrhage |  |  | V |
| Renal cell carcinoma |  |  | V |
| Abnormality of coagulation |  |  | V |
| Abnormal palate morphology |  |  | V |
| Thick eyebrow |  |  | V |
| Epicanthus |  |  | V |
| Glaucoma |  |  | V |
| Joint stiffness |  |  | V |
| Abnormal systemic arterial morphology |  |  | V |
| Abnormality of the musculature of the upper limbs |  |  | V |
| Abdominal pain |  |  | V |
| Delayed speech and language development |  |  | V |
| Morphological abnormality of the gastrointestinal tract |  |  | V |
| Thin corpus callosum |  |  | V |
| Palmoplantar blistering |  |  | V |
| Reticulocyte count |  |  | V |
| Aplasia/hypoplasia involving bones of the extremities |  |  | V |
| Abnormal renal cortex morphology |  |  | V |
| Abnormality of digestive system morphology |  |  | V |
| Abnormal thumb morphology |  |  | V |
| Involuntary movements |  |  | V |
| Abnormality of vision |  |  | V |
| Platelet component distribution width |  |  | V |
| Kyphosis |  |  | V |
| Renal neoplasm |  |  | V |
| Seizure |  |  | V |
| Visual impairment |  |  | V |
| Abnormal external genitalia |  |  | V |
| Abnormality of the genital system |  |  | V |
| Abnormal labia morphology |  |  | V |
| Abnormality of male external genitalia |  |  | V |
| Abnormal emotion/affect behaviour |  |  | V |
| Chronic infection |  |  | V |
| Abnormality of the common coagulation pathway |  |  | V |
| Abnormal cerebral subcortex morphology |  |  | V |
| Abnormal facial skeleton morphology |  |  | V |
| Abnormal sternum morphology |  |  | V |
| Motor stereotypy |  |  | V |
| Aplasia/Hypoplasia of the corpus callosum |  |  | V |
| Abnormal corpus callosum morphology |  |  | V |
| Wide nasal bridge |  |  | V |
| Depressed nasal bridge |  |  | V |
| Abnormal nasal bridge morphology |  |  | V |
| Abnormal nasal morphology |  |  | V |
| Abnormal aortic morphology |  |  | V |
| Abnormality of the nose |  |  | V |
| Language impairment |  |  | V |
| Abnormality of the cervical spine |  |  | V |
| Abnormality of muscle size |  |  | V |
| Lipodystrophy |  |  | V |
| Leukoencephalopathy |  |  | V |
| Peripheral neuropathy |  |  | V |
| Abnormal respiratory system morphology |  |  | V |
| Abnormal hand morphology |  |  | V |
| Poor speech |  |  | V |
| Absent speech |  |  | V |
| Abnormality of complement system |  |  | V |
| Cleft soft palate |  |  | V |
| Self-injurious behaviour |  |  | V |
| Abnormality of the palpebral fissures |  |  | V |
| Abnormal circulating nitrogen compound concentration |  |  | V |
| Hamartoma |  |  | V |
| Increased circulating lactate dehydrogenase concentration |  |  | V |
| Long eyelashes |  |  | V |
| Palmoplantar keratoderma |  |  | V |
| Micrognathia |  |  | V |
| Abnormal right ventricle morphology |  |  | V |
| Aplasia/Hypoplasia of the mandible |  |  | V |
| Abnormal mandible morphology |  |  | V |
| Atrophy/Degeneration affecting the central nervous system |  |  | V |
| Abnormal peripheral nervous system morphology |  |  | V |
| Amblyopia |  |  | V |
| Ventricular septal defect |  |  | V |
| Abnormality of urine homeostasis |  |  | V |
| Somatic sensory dysfunction |  |  | V |
| Fatigue |  |  | V |
| Ventriculomegaly |  |  | V |
| Cerebral cortical atrophy |  |  | V |
| Abnormal granulocyte count |  |  | V |
| Synostosis involving bones of the upper limbs |  |  | V |
| Gastroesophageal reflux |  |  | V |
| Skeletal muscle atrophy |  |  | V |
| Distal renal tubular acidosis |  |  | V |
| Prominent ear helix |  |  | V |
| Acantholysis |  |  | V |
| Proximal hyperreflexia |  |  | V |
| Tooth malposition |  |  | V |
| Impairment in personality functioning |  |  | V |
| Synostosis of joints |  |  | V |
| Renal hypoplasia/aplasia |  |  | V |
| Abnormality of mouth size |  |  | V |
| Delayed puberty |  |  | V |
| Stereotypical hand wringing |  |  | V |
| Jaundice |  |  | V |
