# Supplementary material for: Placental Protein Citrullination Signatures Are Modified in Early- and Late-Onset Fetal Growth Restriction
Source: Int J Mol Sci. 2025 Apr 29;26(9):4247. doi: 10.3390/ijms26094247 (PMC12071715; doi:10.3390/ijms26094247)
Supplement: Supplementary file 1 [file ijms-26-04247-s001.zip › Supplementary Table S4.docx]

**Supplementary Table S4:** Reactome pathways associated with the placental citrullinomes. A tick (V) indicates that the pathway was present in the placental citrullinome of the group. Control (AGA), early-onset FGR (E-FGR) and late-onset FGR (L-FGR) samples.

| **Reactome Pathway** | **Control AGA** | **E-FGR** | **L-FGR** |
| --- | --- | --- | --- |
| Disease (HSA-1643685) | V |  |  |
| Cytokine (HSA-9678108) | V |  |  |
| HSF1 activation (HSA-3371511) | V |  |  |
| Glycolysis (HSA-70171) | V |  |  |
| snRNP Assembly (HSA-191859) | V |  |  |
| Zinc efflux and compartmentalization by the SLC30 family | V |  |  |
| Purinergic signalling in leishmaniasis infection | V |  |  |
| Oestrogen biosynthesis | V |  |  |
| Metabolism of steroids | V |  |  |
| Attenuation phase | V | V |  |
| Response of Mtb to phagocytosis | V |  | V |
| Metabolism of RNA | V | V | V |
| L13a-mediated translational silencing of Ceruloplasmin expression | V | V | V |
| Peptide chain elongation | V | V | V |
| Influenza Infection | V | V | V |
| Formation of a pool of free 40S subunits | V | V | V |
| Infectious disease | V | V | V |
| Cap-dependent Translation Initiation | V | V | V |
| GTP hydrolysis and joining of the 60S ribosomal subunit | V | V | V |
| Selenocysteine synthesis | V | V | V |
| Nonsense Mediated Decay (NMD) independent of the Exon Junction Complex (EJC) | V | V | V |
| Viral mRNA Translation | V | V | V |
| Regulation of expression of SLITs and ROBOs | V | V | V |
| Nonsense Mediated Decay (NMD) enhanced by the Exon Junction Complex (EJC) | V | V | V |
| Eukaryotic Translation Termination | V | V | V |
| Response of EIF2AK4 (GCN2) to amino acid deficiency | V | V | V |
| Cellular responses to stress | V | V | V |
| Metabolism of proteins | V | V | V |
| SRP-dependent co-translational protein targeting to membrane | V | V | V |
| Axon guidance | V | V | V |
| Influenza Viral RNA Transcription and Replication | V | V | V |
| Cellular response to starvation | V | V | V |
| Major pathway of rRNA processing in the nucleolus and cytosol | V | V | V |
| Metabolism of amino acids and derivatives | V | V | V |
| Translation | V | V | V |
| Developmental Biology | V | V | V |
| Immune System | V | V | V |
| Translation initiation complex formation | V | V | V |
| Neutrophil degranulation | V | V | V |
| Ribosomal scanning and start codon recognition | V | V | V |
| SARS-CoV-1 modulates host translation machinery | V | V | V |
| Metabolism | V | V | V |
| Formation of the ternary complex, and subsequently, the 43S complex | V | V | V |
| SARS-CoV-2 modulates host translation machinery | V | V | V |
| Innate Immune System | V | V | V |
| SARS-CoV-1-host interactions | V | V | V |
| mRNA Splicing - Major Pathway | V | V | V |
| Haemostasis | V | V | V |
| RHO GTPase cycle | V | V | V |
| Processing of Capped Intron-Containing Pre-mRNA | V | V | V |
| Post-translational protein modification | V | V | V |
| Platelet degranulation | V | V | V |
| SARS-CoV Infections | V | V | V |
| SARS-CoV-2-host interactions | V | V | V |
| SARS-CoV-2 Infection | V | V | V |
| Platelet activation, signalling and aggregation | V | V | V |
| Regulation of Insulin-like Growth Factor (IGF) transport and uptake by Insulin-like Growth Factor Binding Proteins (IGFBPs) | V | V | V |
| Post-translational protein phosphorylation | V | V | V |
| RHOQ GTPase cycle | V | V | V |
| Zinc transporters | V | V | V |
| mRNA Splicing - Minor Pathway | V | V | V |
| Cellular response to heat stress | V | V | V |
| Protein methylation | V | V | V |
| Signalling by MET | V | V | V |
| Metabolism of steroid hormones | V | V | V |
| Regulation of HSF1-mediated heat shock response | V | V | V |
| Chaperone Mediated Autophagy | V | V | V |
| Signalling by ROBO receptors |  | V | V |
| Cellular responses to stimuli |  | V | V |
| Selenoamino acid metabolism |  | V | V |
| Eukaryotic Translation Elongation |  | V | V |
| Disease |  | V | V |
| Signalling by Rho GTPases |  | V | V |
| SARS-CoV-1 Infection |  | V | V |
| SARS-CoV-1 targets host intracellular signalling and regulatory pathways |  | V | V |
| RHO GTPase Effectors |  | V | V |
| Interleukin-12 family signalling |  | V | V |
| Signalling by Interleukins |  | V | V |
| Vesicle-mediated transport |  | V | V |
| Diseases of signal transduction by growth factor receptors and second messengers |  | V | V |
| Gene and protein expression by JAK-STAT signalling after Interleukin-12 stimulation |  | V | V |
| Apoptosis |  | V | V |
| Cytokine Signalling in Immune system |  | V | V |
| Scavenging of heme from plasma |  | V | V |
| Signal Transduction |  | V | V |
| Regulation of mRNA stability by proteins that bind AU-rich elements |  | V | V |
| TCF dependent signalling in response to WNT |  | V | V |
| Signalling by WNT |  | V | V |
| CDC42 GTPase cycle |  | V | V |
| AUF1 (hnRNP D0) binds and destabilizes mRNA |  | V | V |
| Binding and Uptake of Ligands by Scavenger Receptors |  | V | V |
| Cellular response to chemical stress |  | V | V |
| RAC1 GTPase cycle |  | V | V |
| Extracellular matrix organization |  | V | V |
| ER-Phagosome pathway |  | V | V |
| PCP/CE pathway |  | V | V |
| Erythrocytes take up carbon dioxide and release oxygen |  | V | V |
| Signalling by RAF1 mutants |  | V | V |
| Formation of the cornified envelope |  | V | V |
| RHOJ GTPase cycle |  | V | V |
| Signalling by ALK fusions and activated point mutants |  | V | V |
| Host Interactions of HIV factors |  | V | V |
| Degradation of beta-catenin by the destruction complex |  | V | V |
| Signalling by moderate kinase activity BRAF mutants |  | V | V |
| Paradoxical activation of RAF signalling by kinase inactive BRAF |  | V | V |
| Signalling downstream of RAS mutants |  | V | V |
| RAC2 GTPase cycle |  | V | V |
| M Phase |  | V | V |
| Signalling by high-kinase activity BRAF mutants |  | V | V |
| Transport of small molecules |  | V | V |
| Nuclear events mediated by NFE2L2 |  | V | V |
| Beta-catenin independent WNT signalling |  | V | V |
| MAPK family signalling cascades |  | V | V |
| Membrane Trafficking |  | V | V |
| UCH proteinases |  | V | V |
| Signalling by BRAF and RAF1 fusions |  | V | V |
| Cell-extracellular matrix interactions |  | V | V |
| Negative regulation of NOTCH4 signalling |  | V | V |
| RHO GTPases activate KTN1 |  | V | V |
| Degradation of DVL |  | V | V |
| RAF/MAP kinase cascade |  | V | V |
| Factors involved in megakaryocyte development and platelet production |  | V | V |
| MAPK6/MAPK4 signalling |  | V | V |
| Deubiquitination |  | V | V |
| Defective CFTR causes cystic fibrosis |  | V | V |
| EPH-Ephrin signalling |  | V | V |
| Ub-specific processing proteases |  | V | V |
| ABC transporter disorders |  | V | V |
| RAC3 GTPase cycle |  | V | V |
| Transcriptional regulation by RUNX3 |  | V | V |
| Metal ion SLC transporters |  | V | V |
| RHOA GTPase cycle |  | V | V |
| Cross-presentation of soluble exogenous antigens (endosomes) |  | V | V |
| Regulation of activated PAK-2p34 by proteasome mediated degradation |  | V | V |
| Hedgehog ligand biogenesis |  | V | V |
| Regulation of ornithine decarboxylase (ODC) |  | V | V |
| RHOV GTPase cycle |  | V | V |
| Vpu mediated degradation of CD4 |  | V | V |
| Autodegradation of the E3 ubiquitin ligase COP1 |  | V | V |
| Ubiquitin Mediated Degradation of Phosphorylated Cdc25A |  | V | V |
| Ubiquitin-dependent degradation of Cyclin D |  | V | V |
| GSK3B and BTRC:CUL1-mediated-degradation of NFE2L2 |  | V | V |
| Apoptotic cleavage of cellular proteins |  | V | V |
| RHOD GTPase cycle |  | V | V |
| Vif-mediated degradation of APOBEC3G |  | V | V |
| Regulation of RUNX3 expression and activity |  | V | V |
| SCF-beta-TrCP mediated degradation of Emi1 |  | V | V |
| L1CAM interactions |  | V | V |
| Degradation of AXIN |  | V | V |
| FBXL7 down-regulates AURKA during mitotic entry and in early mitosis |  | V | V |
| Cell Cycle, Mitotic |  | V | V |
| Hh mutants are degraded by ERAD |  | V | V |
| MET promotes cell motility |  | V | V |
| Cell Cycle |  | V | V |
| Antigen Presentation: Folding, assembly and peptide loading of class I MHC |  | V | V |
| EPHB-mediated forward signalling |  | V | V |
| Laminin interactions |  | V | V |
| RHOC GTPase cycle |  | V | V |
| NIK-->noncanonical NF-kB signalling |  | V | V |
| SCF(Skp2)-mediated degradation of p27/p21 |  | V | V |
| Non-integrin membrane-ECM interactions |  | V | V |
| Dectin-1 mediated noncanonical NF-kB signalling |  | V | V |
| Degradation of GLI1 by the proteasome |  | V | V |
| Degradation of GLI2 by the proteasome |  | V | V |
| GLI3 is processed to GLI3R by the proteasome |  | V | V |
| RHO GTPases activate IQGAPs |  | V | V |
| Assembly of the pre-replicative complex |  | V | V |
| The role of GTSE1 in G2/M progression after G2 checkpoint |  | V | V |
| Autodegradation of Cdh1 by Cdh1:APC/C |  | V | V |
| Asymmetric localization of PCP proteins |  | V | V |
| Recycling pathway of L1 |  | V | V |
| RUNX1 regulates transcription of genes involved in differentiation of HSCs |  | V | V |
| Oxygen-dependent proline hydroxylation of Hypoxia-inducible Factor Alpha |  | V | V |
| Activation of NF-kappaB in B cells |  | V | V |
| RHO GTPases Activate WASPs and WAVEs |  | V | V |
| ABC-family proteins mediated transport |  | V | V |
| APC/C:Cdc20 mediated degradation of Securin |  | V | V |
| Regulation of RAS by GAPs |  | V | V |
| Regulation of PTEN stability and activity |  | V | V |
| Signalling by Receptor Tyrosine Kinases |  | V | V |
| Peptide hormone metabolism |  | V | V |
| Clathrin-mediated endocytosis |  | V | V |
| Orc1 removal from chromatin |  | V | V |
| Cdc20:Phospho-APC/C mediated degradation of Cyclin A |  | V | V |
| CDK-mediated phosphorylation and removal of Cdc6 |  | V | V |
| Regulation of RUNX2 expression and activity |  | V | V |
| G2/M Checkpoints |  | V | V |
| APC/C:Cdh1 mediated degradation of Cdc20 and other APC/C:Cdh1 targeted proteins in late mitosis/early G1 |  | V | V |
| Adaptive Immune System |  | V | V |
| Disorders of transmembrane transporters |  | V | V |
| ECM proteoglycans |  | V | V |
| MET activates PTK2 signalling |  | V | V |
| Degradation of the extracellular matrix |  | V | V |
| FCERI mediated NF-kB activation |  | V | V |
| Hedgehog on state |  | V | V |
| IRE1alpha activates chaperones |  | V | V |
| Breakdown of the nuclear lamina |  | V | V |
| Apoptotic cleavage of cell adhesion proteins |  | V | V |
| Signalling by the B Cell Receptor (BCR) |  | V | V |
| Mitotic Prophase |  | V | V |
| HSF1-dependent transactivation |  | V |  |
| COPII-mediated vesicle transport |  | V |  |
| Sensory processing of sound |  | V |  |
| Class I MHC mediated antigen processing & presentation |  | V |  |
| Golgi Cisternae Pericentriolar Stack Reorganization |  | V |  |
| Interleukin-4 and Interleukin-13 signaling |  | V |  |
| Sensory processing of sound by outer hair cells of the cochlea |  | V |  |
| Amyloid fiber formation |  | V |  |
| Muscle contraction |  | V |  |
| GPVI-mediated activation cascade |  | V |  |
| Meiotic synapsis |  | V |  |
| Meiosis |  | V |  |
| Metabolism of Angiotensinogen to Angiotensins |  | V |  |
| Scavenging by Class A Receptors |  | V |  |
| Transcriptional regulation by RUNX2 |  | V |  |
| Interleukin-12 signalling |  |  | V |
| Erythrocytes take up oxygen and release carbon dioxide |  |  | V |
| RHOF GTPase cycle |  |  | V |
| RMTs methylate histone arginines |  |  | V |
| Transcriptional regulation by RUNX1 |  |  | V |
| Deregulated CDK5 triggers multiple neurodegenerative pathways in Alzheimer’s disease models |  |  | V |
| Asparagine N-linked glycosylation |  |  | V |
| Nervous system development |  |  | V |
| Regulation of Apoptosis |  |  | V |
| Apoptotic execution phase |  |  | V |
| MAP2K and MAPK activation |  |  | V |
| RHO GTPases Activate Formins |  |  | V |
| Detoxification of Reactive Oxygen Species |  |  | V |
| GRB2:SOS provides linkage to MAPK signalling for Integrins |  |  | V |
| p130Cas linkage to MAPK signalling for integrins |  |  | V |
| Mitotic Anaphase |  |  | V |
| Golgi-to-ER retrograde transport |  |  | V |
| RAB geranylgeranylation |  |  | V |
| ER to Golgi Anterograde Transport |  |  | V |
| G2/M Transition |  |  | V |
| COPI-mediated anterograde transport |  |  | V |
| MHC class II antigen presentation |  |  | V |
| HSP90 chaperone cycle for steroid hormone receptors (SHR) in the presence of ligand |  |  | V |
| Loss of MECP2 binding ability to 5mC-DNA |  |  | V |
| Diseases of programmed cell death |  |  | V |
| Leishmania infection |  |  | V |
| HIV Infection |  |  | V |
| Folding of actin by CCT/TriC |  |  | V |
| The citric acid (TCA) cycle and respiratory electron transport |  |  | V |
| Regulation of actin dynamics for phagocytic cup formation |  |  | V |
| Citric acid cycle (TCA cycle) |  |  | V |
| SARS-CoV-2 targets host intracellular signalling and regulatory pathways |  |  | V |
| RHOBTB GTPase Cycle |  |  | V |
| Mitochondrial protein import |  |  | V |
| RHOBTB2 GTPase cycle |  |  | V |
| Separation of Sister Chromatids |  |  | V |
| Translocation of SLC2A4 (GLUT4) to the plasma membrane |  |  | V |
| Cell-Cell communication |  |  | V |
| Syndecan interactions |  |  | V |
| Prefoldin mediated transfer of substrate to CCT/TriC |  |  | V |
| Signalling by TGF-beta Receptor Complex |  |  | V |
| Cell junction organization |  |  | V |
| Smooth Muscle Contraction |  |  | V |
| Formation of the beta-catenin:TCF transactivating complex |  |  | V |
| Intra-Golgi and retrograde Golgi-to-ER traffic |  |  | V |
| Cell Cycle Checkpoints |  |  | V |
| Signalling by NOTCH |  |  | V |
| FCGR3A-mediated phagocytosis |  |  | V |
| Initiation of Nuclear Envelope (NE) Reformation |  |  | V |
| COPI-dependent Golgi-to-ER retrograde traffic |  |  | V |
| RHO GTPases activate PKNs |  |  | V |
| MECP2 regulates transcription of neuronal ligands |  |  | V |
| Nuclear events stimulated by ALK signalling in cancer |  |  | V |
| HCMV Early Events |  |  | V |
| FOXO-mediated transcription |  |  | V |
| Mitotic G1 phase and G1/S transition |  |  | V |
| Assembly of Viral Components at the Budding Site |  |  | V |
| N-glycan trimming in the ER and Calnexin/Calreticulin cycle |  |  | V |
| RHO GTPases activate PAKs |  |  | V |
| ATF6 (ATF6-alpha) activates chaperone genes |  |  | V |
| Zinc influx into cells by the SLC39 gene family |  |  | V |
| G1/S Transition |  |  | V |
| Hedgehog off state |  |  | V |
| Pyruvate metabolism and Citric Acid (TCA) cycle |  |  | V |
| Downstream TCR signalling |  |  | V |
| Unfolded Protein Response (UPR) |  |  | V |
| RHOBTB1 GTPase cycle |  |  | V |
| TCR signalling |  |  | V |
| Formation of Fibrin Clot (Clotting Cascade) |  |  | V |
| Vpr-mediated induction of apoptosis by mitochondrial outer membrane permeabilization |  |  | V |
| Insulin-like Growth Factor-2 mRNA Binding Proteins (IGF2BPs/IMPs/VICKZs) bind RNA |  |  | V |
